# The Body Status of Manganese and Activity of This Element-Dependent Mitochondrial Superoxide Dismutase in a Rat Model of Human Exposure to Cadmium and Co-Administration of *Aronia melanocarpa* L. Extract

**DOI:** 10.3390/nu14224773

**Published:** 2022-11-11

**Authors:** Małgorzata M. Brzóska, Małgorzata Gałażyn-Sidorczuk, Magdalena Kozłowska, Nazar M. Smereczański

**Affiliations:** Department of Toxicology, Medical University of Bialystok, Adama Mickiewicza 2C Street, 15-222 Bialystok, Poland

**Keywords:** apparent absorption, *Aronia melanocarpa* L. berries extract, body status, cadmium, chokeberry extract, manganese, manganese-dependent superoxide dismutase, mitochondria, oxidative/antioxidative balance, polyphenols

## Abstract

The impact of a polyphenol-rich 0.1% aqueous extract from *Aronia melanocarpa* L. berries (AE) on the body status of manganese (Mn) and the activity of this essential element-dependent mitochondrial superoxide dismutase (MnSOD) during treatment with cadmium (Cd) was investigated in a rat model of low-level and moderate environmental human exposure to this xenobiotic (1 and 5 mg Cd/kg diet, respectively, for 3–24 months). The exposure to Cd, dose- and duration-dependently, affected the body status of Mn (apparent absorption, body retention, serum and tissue concentrations, content in some organs and total Mn body burden, and urinary and faecal excretion) and the activity of MnSOD in the mitochondria of the liver, kidney, and brain. The administration of AE during the exposure to Cd prevented or at least partially protected the animals from the perturbation of the metabolism of Mn, as well as ameliorated changes in the activity of MnSOD and the concentration of Mn and protected from Cd accumulation in the mitochondria. In conclusion, AE may protect from disorders in the body status of Mn and influence the antioxidative capacity of cells under chronic exposure to Cd. The findings confirm the protective impact of aronia berries products against Cd toxicity.

## 1. Introduction

It is well known that the mineral status of the body has a great impact on health and thus a growing interest has been observed in the recognition of factors that may perturb the metabolism and function of essential elements [1,2,3]. Moreover, not only a deficiency of any bioelement but also its excess are unfavourable for health [2,3,4,5]. One of these elements is manganese (Mn). Mn is an essential trace element in living organisms and both its deficiency and excess in the body may be dangerous [4,5,6,7,8]. The maintenance of the body’s homeostasis of this element is necessary for the proper functioning of numerous processes in the organism, including the detoxification of reactive oxygen species (ROS) [9,10,11,12,13,14,15]. Divalent Mn (Mn(II)) as a co-factor of mitochondrial superoxide dismutase (MnSOD) scavenges oxygen free radicals (superoxide radical–O_2_**^·^**^−^ and hydroxyl radical –**^·^**OH) and thus plays an important role in maintaining oxidative/antioxidative balance and protecting from the development of oxidative stress and its consequences [13,14]. It has been revealed that Mn(II) administered at low single doses acts as a potent antioxidant in experimental animals [9,10,11], whereas its higher repeated intake may cause neurodegenerative changes in children and animals [4,5,6,8]. Moreover, the available literature data [9,10,11,16] show that Mn(II) at low concentrations/doses may protect against the toxic action of cadmium (Cd) due to its antioxidative properties and ability to inhibit the uptake of this heavy metal into the cells.

Nowadays, human exposure to Cd in industrialized countries is inevitable because of contamination of the natural environment and food, and sometimes also of the workplace [17,18]. Epidemiological studies provide evidence that current environmental exposure to Cd may contribute to damage to various organs and systems [17,18,19,20,21]. It is well known that oxidative stress and interactions with essential elements, including Mn, belong to the main mechanisms of the toxic action of Cd [9,10,17,18,22,23,24,25,26,27]. High and moderate exposure to Cd has been reported to perturb the metabolism of Mn in the body [9,10,12,28,29,30]; however, the impact of low-level treatment with this toxic heavy metal on the body status of this essential element has not been studied until now. Owing to the forecasts that exposure to Cd will elevate and the growing number of evidence that even low-level long-lasting intoxication with this element may pose a risk to health [17,18,19,20,21], it is very important, from a public health point of view, to find effective nutritional agents to prevent against harmful effects of exposure to this xenobiotic.

The results of our previous studies indicate that the polyphenol-rich berries of *Aronia melanocarpa* L. (*Michx.*, Elliott, *Rosaceae*), also called black chokeberries, seem to be very promising agents in the protection from unfavourable effects of exposure to Cd. We have revealed, in the rat model of low-level and moderate human exposure to Cd during a lifetime in industrialized countries (1 and 5 mg Cd/kg diet, respectively, for up to 24 months), that the administration of 0.1% aqueous extract from the berries of *A. melanocarpa* (AE) reduced the absorption of Cd from the gastrointestinal tract (by 14–17%) and its accumulation in the body [31] and protected from bone damage [22,32,33], liver damage [23,24,34], and submandibular glands damage [25], as well as from perturbing the metabolism of zinc (Zn) and copper (Cu) [26]. The exposure to this xenobiotic dose- and duration dependently influenced the gastrointestinal absorption, retention in the body, concentrations in the serum and tissues, content in internal organs, and excretion from the body of Zn and Cu, as well as decreased the amount of these elements unbound to metallothionein (MT). The low-level lifetime intoxication with this toxic metal, unlike the moderate treatment, resulted in an increase in the total content of Zn and Cu in the liver and/or kidney, while the co-administration of AE protected (completely or partially) against most of the Cd-induced changes in the metabolism of these elements [26]. We are continuing our research to explain whether this extract may also protect from other unfavourable outcomes of action of this heavy metal and to clarify the possible mechanisms of this protection. Only a complex evaluation of the impact of the chokeberry extract performed in the same model will allow recognition of whether the extract may be a good candidate to be a potentially effective strategy to counteract the harmful outcomes of environmental intoxication with this toxic metal.

Because the administration of the 0.1% AE decreased the body burden of Cd [31], we have hypothesized that the extract could also, at least partially, protect from this heavy metal-induced disturbance in the body status of Mn and in this way influence the activity of this element dependent potent antioxidative enzyme–MnSOD in the mitochondria of cells. However, taking into consideration that ingredients of AE may potentially form complexes with ions of Mn (Mn^2+^) [35,36], it cannot be ruled out that the extract administration may lead to this element deficiency in the body or have no influence on its status. That is why the present study aimed to investigate the impact of the administration of the AE on the body status of Mn and its involvement in the maintenance of the antioxidative protection in the mitochondria, which due to high oxygen consumption constitute a major source of ROS in cells and are especially susceptible to damage by pro-oxidants via the oxidative stress-related mechanisms [14,15,20,37]. For this purpose, the apparent absorption, retention in the body, and excretion of Mn, as well as the serum and tissue concentrations of this element, and its total content in internal organs were estimated. Moreover, the activity of MnSOD, as well as the concentrations of Mn and Cd in the mitochondrial fraction of the liver and kidney, were assayed, as the main organs of Cd accumulation in the body [31], as well as in the brain, which due to the abundance of lipids and high oxygen metabolism, is especially susceptible to damage by pro-oxidants [21,38]. We have expected that this study will provide new and practically useful data on the risk of perturbing the body status of Mn due to exposure to Cd and on the possibility of the use of aronia berries products in the protection from Cd-induced changes in the metabolism of this essential bioelement.

## 2. Materials and Methods

### 2.1. Chemicals

Cadmium chloride (CdCl_2_ × 2.5 H_2_O; purity > 99.5%), sodium chloride (NaCl), dipotassium hydrogen phosphate, and potassium dihydrogen phosphate were provided by POCh (Gliwice, Poland). Heparin was purchased from Biochemie GmbH (Kundl, Austria), while Morbital and Triton X-100 were obtained from Biowet (Pulawy, Poland) and Sigma-Aldrich (St. Louis, MO, USA), respectively. Butylated hydroxytoluene was provided by Sigma-Aldrich (St. Louis, MO, USA). Trace-pure concentrated hydrochloric acid (HCl, 30%, trace metal grade) and nitric acid (HNO_3_, 65%, trace metal grade), obtained from Merck (Darmstadt, Germany), and stocks of standard solutions of Mn and Cd assigned for atomic absorption spectrometry (AAS method), purchased from C.P.A. Ltd. (Stara Zagora, Bulgaria), were used. The mixture of palladium and magnesium (as nitrates; C.P.A. Ltd. Stara Zagora, Bulgaria) was used as a matrix modifier in Mn and Cd analysis. To check the analytical quality of metal measurements, the following certified reference materials were used: Bovine Liver (no. 1577b; National Institute of Standards and Technology, Gaithersburg, MD, USA), Pig Kidney (BCR-186; Institute for Reference Materials and Measurements, Geel, Belgium), Trace Elements Serum L-1 LOT (no. 0903106; SERO AS, Billingstad, Norway), Trace Elements Urine L-2 LOT (no. 1011645; SERO AS; Billingstad, Norway), and Bone Ash (no. 1400; National Institute of Standards and Technology, Gaithersburg, MD, USA). Ultra-pure water, received from compact water purification system Select HP 40 (Purite Ltd., Thame, Oxfordshire, UK), was used.

The Superoxide Dismutase Assay Kit (no. 706002), for the determination of the activity of MnSOD, was obtained from Cayman Chemical Company (Ann Arbor, MI, USA), whereas the diagnostic kit (no. 1-055-0200) for total protein assay was obtained from BioMaxima (Lublin, Poland).

### 2.2. Experimental Animals

**Figure 1:**
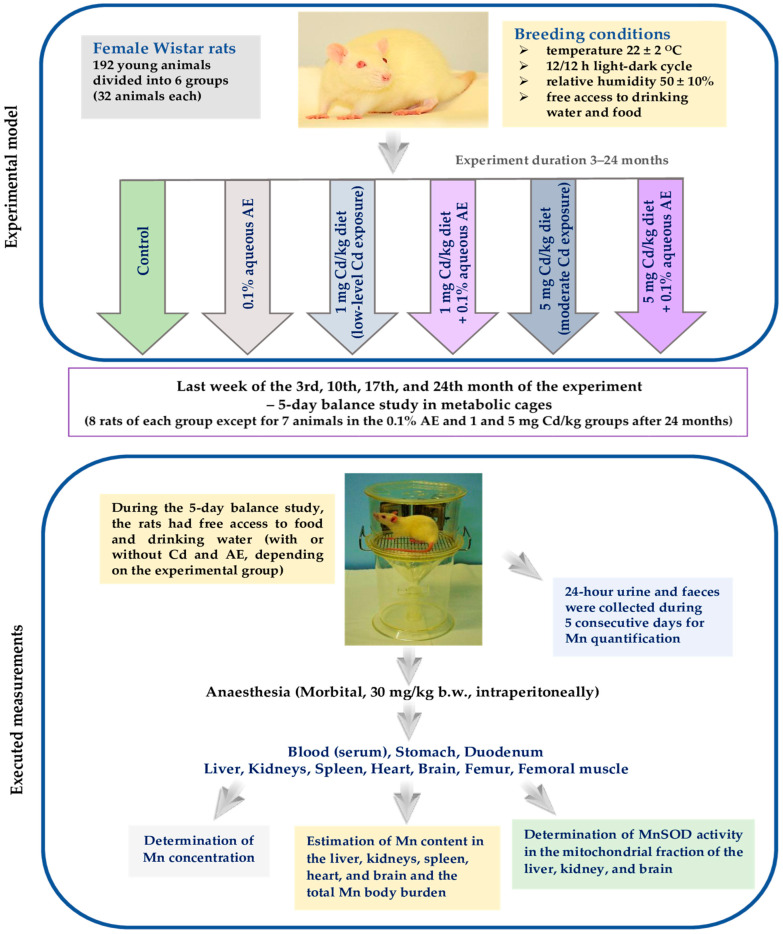
Schematic representation of the experimental model used in the present study and the executed measurements. This experimental model has already been described in detail [22,23,24,26,31,32,33,34]. AE, extract from the berries of *Aronia melanocarpa* L.; Cd, cadmium; Mn, manganese; MnSOD, manganese-dependent superoxide dismutase.

The study was performed on 192 young (three- to four-week-old) female Wistar rats (Hannover Wistar rats bred using the Charles River International Genetic Standardization Program–Crl: WI (Han)) coming from the certified Laboratory Animal House (Brwinów, Poland). The animals were maintained in stainless-steel cages (four rats in each) under controlled conventional conditions (Figure 1). During the first 3 months of the study, all females were fed with the Labofeed H diet (breeding diet ensuring proper growth and development of young animals; Label Food “Morawski”, Kcynia, Poland) containing, according to the manufacturer, 145 mg Mn/kg. From the 4th month of the study to its end, the animals received the Labofeed B diet (maintenance diet) that contained 120 mg Mn/kg (manufacturer’s data).

### 2.3. Experimental Protocol

The research protocol was approved by the Local Ethics Committee for Animal Experiments in Bialystok (approval no. 60/2009 on 21 September 2009). The experiment was conducted according to the ethical principles and institutional guidelines, as well as the International Guide for the Use of Animals in Biomedical Research.

Because the experimental model used in the current study has been reported in detail in our previous articles [22,23,24,26,31,32,33,34], in the present paper it is described only briefly and presented schematically in Figure 1.

The rats were randomly divided into six equal groups (mean body weight of animals about 65 g) and administered a diet containing Cd (1 or 5 mg Cd/kg) and/or 0.1% aqueous AE (used as the only drinking fluid), as described in Figure 1. The animals of the control group drank distilled water without the addition of AE and like the rats of the 0.1% AE group were maintained on the standard Labofeed diets without Cd.

The diets containing 1 and 5 mg Cd/kg were prepared by the addition of CdCl_2_ × 2.5 H_2_O into the components of the standard Labofeed H diet and Labofeed B diet at the stage of their production by Label Food “Morawski’’. Mean Cd concentration in these diets quantified in our laboratory agreed with the certified values and reached 1.09 ± 0.13 mg/kg (mean ± standard deviation–SD) and 4.92 ± 0.53 mg/kg, respectively, whereas its concentration in the standard Labofeed diets was 0.0584 ± 0.0049 mg/kg [31]. The diets containing 1 and 5 mg Cd/kg were used to create the conditions of rats’ exposure to this toxic metal reflecting current low-level and moderate, respectively, repeated human exposure to this heavy metal in industrialized countries. The determination of the concentration of Cd in the urine and blood (markers of exposure) of the animals maintained on the diets containing 1 and 5 mg Cd/kg alone or together with AE (0.0852–0.2762 μg/g creatinine and 0.103–0.306 μg/L, and 0.2839–0.8197 μg/g creatinine and 0.584–1.332 μg/L, respectively) [31] confirmed that the experimental model well reflects environmental intoxication with this xenobiotic in economically developed countries [17,19,21].

The 0.1% AE was prepared from the lyophilized powdered chokeberry extract, provided by Adamed Consumer Healthcare (Tuszyn, Poland), by dissolving in redistilled water. According to the manufacturer’s declaration (Certificate KJ 4/2010), the extract contained 65.74% of polyphenolic compounds, including 18.65% of anthocyanins. The following amounts of these compounds (mean ± standard error–SE) were quantified in 1 mL of the 0.1% extract: total polyphenols–612.40 ± 3.33 ng, total anthocyanins–202.28 ± 1.28 ng, cyanidin derivatives such as cyanidin 3-O-β-galactoside–80.07 ± 1.05 ng, cyanidin 3-O-α-arabinoside–33.21 ± 0.01 ng, and cyanidin 3-O-β-glucoside–3.68 ± 0.01 ng, total proanthocyanidins–129.87 ± 1.12 ng, total phenolic acids–110.92 ± 0.89 ng, and chlorogenic acid–68.32 ± 0.08 ng, as well as total flavonoids–21.94 ± 0.98 ng [32]. Other components such as carotenoids, pectins, sugar, sugar alcohols (parasorboside and sorbitol), phytosterols, triterpenes, β-carotene, dietary fiber, tannins, organic acids (L-malic acid, citric acid), carbohydrates, proteins, vitamins (from group B, vitamins C, E, and K), and minerals (calcium, magnesium, and iron) are also present in the extract ([39], producer data). The concentration of Cd in the 0.1% AE was below the limit of detection (<0.05 ng/mL) [31].

The administration of AE in the form of 0.1% aqueous solution allowed to reach in animals the daily intake of polyphenols several times higher, but not too high (Table 1), than their average intake in the general population. The intake of polyphenols in humans all over the world has been estimated at 1000 mg/24 h on average (14.29 mg/kg of body weight–b.w. assuming the average body weight as 70 kg) and ranges from about 800 mg/24 h to above 1700 mg/24 h [40,41,42,43].

As is evident from the data provided in Table 1, throughout the experiment there were no differences in the intakes of Cd and AE regardless of whether they were administered alone or in conjunction. There were also no differences in the consumption of food and drinking water or body weight gain among the experimental groups [31]. No unfavourable health outcomes were observed; however, one animal from the AE group and groups exposed to Cd at the concentration of 1 and 5 mg/kg diet died between the 18th and 24th months of the experiment [31].

In the last week of the 3rd, 10th, 17th, and 24th month of the experiment, the animals (8 or 7 rats of each group) were placed individually in metabolic cages for 24-h urine and faeces collection during a 5-day balance study (Figure 1). The urine and faeces were removed from the metabolic cages every 24 h and stored for further analysis. Immediately after collection, the urine was centrifuged (MPW-350R centrifugator, Medical Instruments, Warsaw, Poland) and its volume was recorded. After the balance study, the animals were fasted overnight and then subjected to anaesthesia under which the whole blood was taken by cardiac puncture with and without anticoagulant (heparin) and various organs and tissues were dissected (Figure 1). The content of the stomach and duodenum was immediately removed by multiple rinsing with ice-cold 0.9% NaCl (physiological saline). The liver, kidneys, spleen, heart, brain, and femoral muscle, after rinsing with ice-cold physiological saline, were gently dried on the filter paper. The femurs were cleaned of all adherent soft tissues. Next, all dissected organs and tissues were weighed with an analytical balance (OHAUS^®^, Nanikon, Switzerland; accuracy to 0.0001 g). The biological material which was not used immediately was stored frozen (−70 °C) until assayed.

### 2.4. Assay of the Concentration of Mn in Biological Fluids, Tissues, and Faeces, as Well as in the Labofeed Diets, Redistilled Water, and 0.1% AE

Slices of wet tissues (liver, left kidney, spleen, heart, brain, femoral muscle, stomach, and duodenum) of all survived rats, weighing about 0.2 g (weighted with accuracy to 0.0001 g), were subjected to digestion with trace-pure 65% HNO_3_ and 30% HCl (both trace metal grade; 9:1 ratio, volume/volume–*v/v*) as reported [31]. The 5-day faeces, after drying (at 110 °C) with the use of a laboratory incubator (Incucenter IC80, SalvisLab, Rotkreuz, Switzerland) to constant weight, was crumbled and for each animal, three representative samples (0.2 g) were collected for analysis and were wet digested with a mixture of HNO_3_ and HCl similarly as soft tissues. The wet mineralization was performed in a microwave system (Multiwave, Anton Paar GmbH, Graz, Austria). After complete digestion and cooling, the samples were transferred into quartz crucibles and placed on the ceramic-glass heater (type PV 300, FALC Instruments, Treviglio, Italy) located under the fume cupboard (Köttermann, type 454-CAND, Uetze/Hänigsen, Germany) to evaporate the remained acids during slight warming up until the solution was completely clear. Next, the preparations were cooled to room temperature and transferred quantitatively, with ultra-pure water, to glass tubes and the samples were adjusted to the final volume of 10 mL. Bone slices (0.1–0.2 g) obtained from the distal epiphysis (trabecular bone region) and diaphysis (compact bone region) of the femur (left), after rinsing with ultra-pure water (to eliminate the remove-available bone marrow), were dried (to receive dry bone weight) and ashed in an electric oven (Muffle Furnace Nabertherm L9/11SKM, Lilienthal, Germany) as reported [31]. The received bone ash was digested with trace-pure 65% HNO_3_, and then the specimens were adjusted to 5 mL, with ultra-pure water. The samples of the serum and representative samples of the urine collected during the 5-day balance study were diluted (according to the concentration range) with 0.5% (*v*/*v*) HNO_3_ containing an addition of Triton X-100 (100 μL of Triton X-100 were used per 100 mL of 0.5% HNO_3_ to reduce surface tension). Samples of the Labofeed H and B diets (three representative samples of 0.2 g each), after crumbling, were prepared for Mn analysis like the soft tissues and faeces.

The concentration of Mn in such preparations of the serum, urine, faeces, soft tissues, bone tissue, and diet, as well as in the samples of distilled water and 0.1% AE, was determined by flameless AAS method with electrothermal atomization in a graphite furnace (GF AAS). The measurements were carried out using a high-resolution continuum source atomic absorption spectrometer ContrAA^®^ 700 (Analytik Jena AG, Jena, Germany) equipped with a transversely heated graphite tube atomizer and graphite tube autosampler MPE. Five μL of the mixture of palladium nitrate and magnesium nitrate, as a matrix modifier, were automatically added to each analyzed sample (20 μL). The primary analytical line at 279.4817 nm was used for Mn determination. The quantification was performed based on the method of the calibration curve. The range of calibration standards for Mn was 0.5–5 μg/L. The standard solutions were prepared with spectroscopically pure water. The limit of detection of Mn was 0.14 μg/L. To keep the concentrations of Mn in analyzed samples within the range of concentrations of the standard curve, the analyzed samples were appropriately diluted if necessary. The concentration of Mn in the soft tissues is expressed in calculation per gram of wet tissue weight, while in the bone tissue–in calculation per gram of dry bone weight.

The accuracy of the measurements was monitored by internal quality control. For quality assurance, the certified reference materials of the serum, liver, kidney, bone, and urine were used (Table S1). The concentration of Mn determined in the certified materials well corresponded with the reference values and the precision of measurements expressed as a coefficient of variation (CV) ranged from 1.8 to 4.1% (detailed results are presented in Table S1). The recovery for Mn was 95–109% (Table S1). Moreover, to avoid accidental contamination of samples, all glassware used under the analysis was treated with 20% HNO_3_ for 24 h and next rinsed with ultra-pure water and all solutions were prepared with the use of spectroscopically pure water.

### 2.5. Evaluation of the Bioavailability and Retention of Mn in the Body

The bioavailability of Mn was evaluated based on this element’s apparent absorption expressed as the index of absorption (Abs_Mn_ [%]) calculated using the following formula: Abs_Mn_ = (I_Mn_ − FE_Mn_)/I_Mn_ × 100%, where I_Mn_ is the mean daily intake of Mn via food during the 5-day balance study and FE_Mn_ is the mean amount of Mn daily excreted with faeces during the 5-day study (expressed as μg Mn/24 h) [26,31].

The mean daily body retention of Mn (R_Mn_ [%]) was calculated as the difference between the mean daily intake of this element (I_Mn_) during the 5-day balance study and the mean amount of the element daily excreted (expressed as μg Mn/24 h) with faeces (FE_Mn_) and urine (UE_Mn_) during this time, according to the following formula: R_Mn_ = [I_Mn_ − (FE_Mn_ + UE_Mn_)]/I_Mn_ × 100% [26,31].

### 2.6. Estimation of the Total Content of Mn in Internal Organs

The total content of Mn in internal organs such as the liver, kidneys, spleen, heart, and brain was evaluated mathematically as the sum of this element content in particular organs [26,31]. The content of Mn in organs was calculated by the multiplication of the concentration of this element in this organ (expressed as μg/g of wet tissue weight) and this organ wet weight (expressed in grams). To evaluate the total content of Mn in both kidneys, it was assumed that its concentration in the right kidney is the same as in the left one.

### 2.7. Determination of the Activity of MnSOD and the Concentrations of Mn and Cd in the Mitochondria of the Liver, Kidney, and Brain

Because MnSOD is present in the mitochondria of the cells [12,20], the activity of this enzyme, as well as the concentrations of Mn and Cd, were determined in the mitochondrial fraction of the liver, kidney, and brain.

#### 2.7.1. Preparation of the Mitochondrial Fraction

Known weight (0.15–0.2 g) slices of the liver, left kidney, and brain (always taken from the same parts of particular organs) were subjected to homogenization in 50 mM potassium phosphate buffer (pH = 7.4; prepared with the use of dipotassium hydrogen phosphate and potassium dihydrogen phosphate) with the addition of butylated hydroxytoluene (10 μL of 0.5 M butylated hydroxytoluene per 1 mL of homogenate was used as an antioxidant) by the usage of a high-performance homogenizer (Ultra-Turrax T25, IKA, Staufen, Germany) to prepare 10% (weight/volume–*w/v*) homogenates. The homogenates were centrifuged (MPW-350R centrifugator, Medical Instruments, Warsaw, Poland) at 1500× *g* for 5 min at 4 °C. Next, the supernatants were centrifuged at 10,000× *g* for 15 min at 4 °C. After the centrifugation, the aliquots were discarded and the received pellets (mitochondrial fraction), after suspension in 50 mM potassium phosphate buffer (pH = 7.4), were divided into two portions. One of them was subjected to the assay of the activity of MnSOD, while in the second the concentrations of Mn and Cd were measured.

#### 2.7.2. Determination of the Activity of MnSOD

Potassium cyanide (2 mM) was added to the portions of the mitochondrial fraction of the liver, kidney, and brain assigned to the assay of MnSOD to inhibit both Cu- and Zn-dependent SOD (CuZnSOD) and extracellular SOD. These allowed the detection of only the activity of MnSOD, which was measured calorimetrically using the Superoxide Dismutase Assay Kit. The assay utilizes a tetrazolium salt for the detection of O_2_^−^ generated by xanthine oxidase and hypoxanthine. The intra-assay CV was <2.3%, whereas the inter-assay CV was <4.9%.

#### 2.7.3. Determination of the Concentrations of Mn and Cd

Known volumes (0.5 mL) of the mitochondrial fractions of the liver, kidney, and brain assigned to the assay of Mn and Cd were wet digested with a mixture of concentrated HNO_3_ and HCl (9:1 *v/v*) using a microwave system (Multiwave, Anton Paar GmbH, Graz, Austria) according to the procedure used in the preparation of soft tissues for the assay of Mn (described in Section 2.4).

The concentrations of Mn and Cd in the wet digests of the mitochondrial fractions of the liver, kidney, and brain were determined by the GF AAS method using a high-resolution continuum source atomic absorption spectrometer ContrAA^®^ 700 (Analytik Jena AG, Jena, Germany) and expressed in calculation per gram of protein. Mn was assayed strictly as reported for its analysis in the wet digests of soft tissues (Section 2.4). The concentration of this element which was simultaneously determined in the Standard Reference Materials (Bovine Liver no. 1577b and Pig Kidney BCR-186) agreed with certified values (Table S1). The recovery was 96–107% and the precision was <4.8% (Table S1).

The primary analytical line at 228.8018 nm was used for Cd determination. Like in the case of the assay of Mn, the mixture of palladium nitrate and magnesium nitrate was used as a matrix modifier. The limit of detection of Cd was 0.015 μg/L. The certified reference materials of the liver and kidney were used to check the analytical quality of Cd determination in the mitochondrial fraction. The concentration of Cd assayed in the certified materials perfectly agreed with the reference values and the precision of measurements (CV) was <6.5%, whereas the recovery was 97–103% (detailed results are presented in Table S1).

#### 2.7.4. Estimation of the Concentration of Total Protein

To adjust the activity of MnSOD and the concentrations of Mn and Cd in the mitochondria of the liver, kidney, and brain, for protein, the concentration of total protein was quantified in the mitochondrial fraction of these organs with the use of the Total Protein BioMaxima S.A. Kit (intra-assay CV < 2.2%).

### 2.8. Statistical Analysis

The data are expressed as a median, 25–75% confidence interval, and minimum and maximum value for eight rats after 3, 10, 17, and 24 months, except for seven animals in the 0.1% AE group and the 1 and 5 mg Cd/kg groups after 24 months and eight to thirty-two animals in the case of the daily intake of Mn throughout the study. Firstly, the Shapiro–Wilk test was conducted for checking the normal distribution of the data. Because the data showed no normal distribution, a nonparametric signed-rank Kruskal–Wallis test was applied to determine whether there were statistically significant (*p* < 0.05) differences among the six experimental groups, and then a Kruskal–Wallis post hoc test was performed for comparison between individual groups and to determine between which two groups a statistically significant (*p* < 0.05) difference occurred. In Tables and Figures, statistically significant differences concerning the control group, the respective group receiving Cd alone (Cd1 + AE vs. Cd1 and Cd5 + AE vs. Cd5), and the respective group exposed to the 1 mg Cd/kg diet alone or with AE (Cd5 vs. Cd1 and Cd5 + AE vs. Cd1 + AE) are marked.

A linear regression analysis was performed to estimate the mutual relationships between the activity of MnSOD and the concentrations of Mn and Cd in the mitochondria of the liver, kidney, and brain. The results of this analysis are presented as a β coefficient (this parameter describes the degree of change in the dependent variable for every 1-unit of change in the independent variable), *R*^2^ (describes what percentage of one variable explains the variability of the other one), and the level of statistical significance (*p*). A dependence between two variables was recognized to be statistically significant at the value of the β coefficient for which *p* < 0.05.

All the statistical calculations were performed using the Statistica 13.3 package (StatSoft, Tulsa, OK, USA).

## 3. Results

### 3.1. Daily Intake of Mn via the Labofeed Diet and Drinking Fluids

The concentration of Mn determined in our laboratory in the Labofeed H and B diets agreed with the certified values provided by the producer and reached 143.8 ± 2.9 mg/kg (mean ± SD) and 117.4 ± 3.4 mg/kg, respectively.

The daily intake of Mn via the Labofeed diets, during the 3, 10, 17, and 24 months of the experiment, as well as the 5-day balance study in the last week of the 3rd, 10th, 17th, and 24th month, did not differ between the experimental groups at particular time points (Table 2 and Table S2). The median daily intake of this element via the Labofeed diets in the control group during the 5-day balance study in the last week of the 3rd, 10th, 17th, and 24th month reached 3.581 mg/24 h (3.379–3.756 mg/24 h), 2.310 mg/24 h (2.208–2.712 mg/24 h), 2.736 mg/24 h (2.388–2.856 mg/24 h), and 3.318 mg/24 h (3.168–3.684 mg/24 h), respectively (Table S2).

The intake of Mn with diet in all groups during the first 3 months of the experiment was higher (1.9–2.3 times) than thereafter (Table 2). The intake of this element in particular experimental groups during the 5-day balance study in the last week of the 3rd month was also higher than after 10 and 17 months (by 31–55%) in all groups, as well as after 24 months in the control, AE, and Cd5 + AE groups (by 8–11%) (Table S2).

The concentration of Mn in distilled water that was administered as the only drinking fluid in the control group and the Cd1 and Cd5 groups and was used to prepare the 0.1% AE was below the limit of detection of the AAS method (0.14 μg/L). Thus, the intake of this element via drinking fluids in these groups was assumed to be 0.

The concentration of Mn determined in the 0.1% AE reached 0.396 ± 0.039 μg/L (mean ± SD). The intake of this essential element via the consumption of the 0.1% AE throughout the 24-month study ranged from 0.0135 to 0.0190 μg/24 h (0.0251–0.0608 μg/kg b.w.), regardless of whether the extract was administered alone or together with Cd (detailed intake of Mn in particular groups is provided in Table S3), and was negligible compared to its intake with the Labofeed diets.

### 3.2. Effect of Cd and/or AE on the Body Status of Mn

#### 3.2.1. Apparent Absorption and the Body Retention of Mn, and Its Faecal and Urinary Excretion

**Figure 2:**
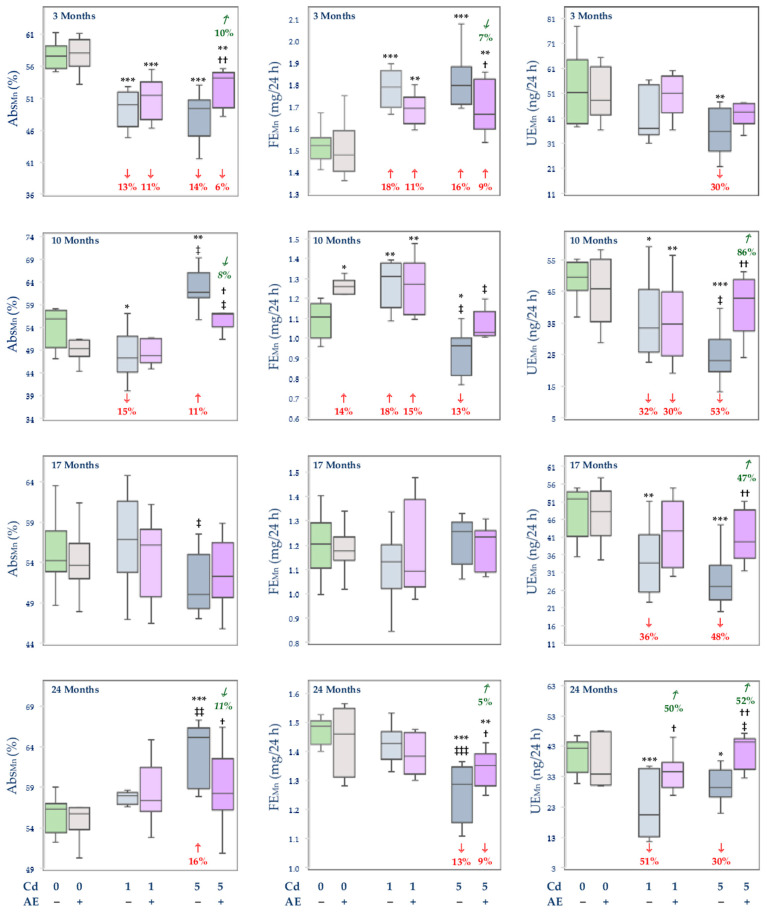
Abs_M_, FE_Mn_, and UE_Mn_ in particular experimental groups. The rats were administered with Cd in the diet at the concentration of 0, 1, and 5 mg/kg and/or 0.1% AE as the only drinking fluid (“+”, administered; “−”, without administration). Data represent a median and 25–75% confidence interval (the horizontal lines within the bars presenting the confidence interval represent median values), as well as the minimum and maximum values for eight rats in each experimental group (except for seven animals in the AE, Cd1, and Cd5 groups after 24 months). Statistically significant differences: * *p* < 0.05, ** *p* < 0.01, *** *p* < 0.001 vs. control group; ^†^ *p* < 0.05, ^††^ *p* < 0.01 vs. respective group receiving Cd alone (Cd1 or Cd5 group); ^‡^ *p* < 0.05, ^‡‡^ *p* < 0.01, ^‡‡‡^ *p* < 0.001 vs. respective group receiving the 1 mg Cd/kg diet alone (Cd1 group) or with the AE (Cd1 + AE group) are marked. The values below and above the bars indicate percentage changes compared to the control group (↓, decrease; ↑, increase) or the respective group receiving Cd alone (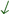, decrease; 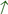, increase), respectively.

Because the Ret_Mn_ in particular experimental groups reached almost the same values as the Abs_Mn_, only the Abs_Mn_ is presented (Figure 2; Ret_Mn_ is shown in Table S4).

The Abs_Mn_ and Ret_Mn_ in the control animals reached 54–58% and were unaffected by the administration of AE alone (Figure 2 and Table S4). The intoxication with the 1 mg Cd/kg diet decreased the Abs_Mn_ and Ret_Mn_ after 3 and 10 months (by 13% and 15%, respectively), while the 10-month co-treatment with AE prevented this effect of Cd (Figure 2 and Table S4). The higher exposure to Cd decreased (by 14%) the Abs_Mn_ and Ret_Mn_ after 3 months and increased the values of these variables after 10 and 24 months (by 11% and 16%, respectively). The simultaneous administration of the extract provided partial (after 3 months) or complete (after 10 and 24 months) protection from the 5 mg Cd/kg diet-mediated alterations in these parameters (Figure 2 and Table S4).

The administration of AE alone had no impact on the FE_Mn_ and UE_Mn_, except for a 14% increase in the FE_Mn_ after 10 months (Figure 2). The low-level exposure to Cd increased the FE_Mn_ after 3 and 10 months (by 18%) and the co-administration of AE did not influence this parameter (Figure 2). The moderate intoxication with this toxic metal led to an increase (by 16%) in the FE_Mn_ after 3 months and a decrease after 10 and 24 months (by 13%) (Figure 2). The co-administration of AE provided partial (3 and 24 months) or complete (10 months) protection against this impact of Cd (Figure 2). The UE_Mn_ was decreased (by 32–51%) from the 10th month in the rats exposed to the 1 mg Cd/kg diet, while the 17- and 24-month co-administration of AE prevented this effect of Cd (Figure 2). In the animals maintained on the diet containing 5 mg Cd/kg, the UE_Mn_ was decreased (by 30–53%) at all-time points and the co-administration of AE entirely protected from the impact of Cd (Figure 2).

The Abs_Mn_ and Ret_Mn_ in the Cd5 group after 10 and 24 months were higher (by 31% and 12%, respectively) than in the Cd1 group, while after 17 months both variables reached lower values (by 12%) at the higher treatment with this xenobiotic (Figure 2 and Table S4). Moreover, the FE_Mn_ after 10 and 24 months and the UE_Mn_ after 10 months of feeding with the diet containing 5 mg Cd/kg were lower (by 27%, 10%, and 31%, respectively) than in the case of the 1 mg Cd/kg diet (Figure 2). In the Cd5 + AE group, the Abs_Mn_ and Ret_Mn_ after 10 months were higher (by 19%) than in the Cd1 + AE group, whereas the FE_Mn_ after 10 months was lower (by 19%) and the UE_Mn_ after 24 months was higher (by 45%) at the moderate exposure (Figure 2 and Table S4).

#### 3.2.2. The Concentration of Mn in the Stomach and Duodenum

**Figure 3:**
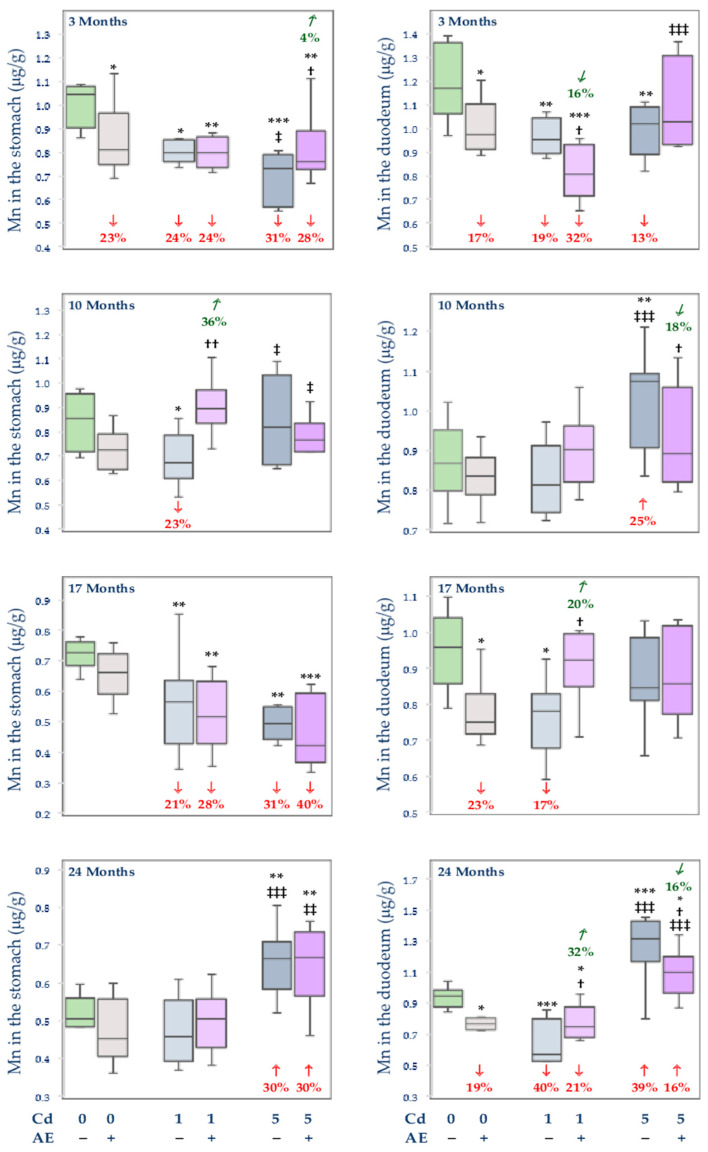
The concentration of Mn in the stomach and duodenum in particular experimental groups. The rats received Cd in the diet at the concentration of 0, 1, and 5 mg/kg and/or 0.1% AE as the only drinking fluid (“+”, administered; “−”, without administration). Data represent a median and 25–75% confidence interval (the horizontal lines within the bars presenting the confidence interval represent median values), as well as the minimum and maximum values for eight rats in each experimental group (except for seven animals in the AE, Cd1, and Cd5 groups after 24 months). Statistically significant differences: * *p* < 0.05, ** *p* < 0.01, *** *p* < 0.001 vs. control group; ^†^ *p* < 0.05, ^††^ *p* < 0.01 vs. respective group receiving Cd alone (Cd1 or Cd5 group); ^‡^ *p* < 0.05, ^‡‡^ *p* < 0.01, ^‡‡‡^ *p* < 0.001 vs. respective group receiving the 1 mg Cd/kg diet alone (Cd1 group) or with the AE (Cd1 + AE group) are marked. The values below and above the bars indicate percentage changes compared to the control group (↓, decrease; ↑, increase) or the respective group receiving Cd alone (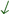, decrease; 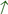, increase), respectively.

The administration of AE alone did not influence the concentration of Mn in the stomach, except for its decrease (by 23%) after 3 months, while the concentration of this element in the duodenum was decreased (by 17–23%) after 3, 17, and 24 months of the study (Figure 3).

The concentration of Mn in the stomach in the Cd1 group was decreased (by 21–24%) for up to 17 months and AE prevented this effect of Cd only after 10 months of co-administration (Figure 3). The moderate intoxication with this xenobiotic decreased (by 31%) the concentration of Mn in the stomach after 3 and 17 months and increased (by 30%) after 24 months (Figure 3). The co-administration of AE partially protected against the 5 mg Cd/kg diet-mediated change in the concentration of Mn only after 3 months of the investigation (Figure 3).

The feeding with the diet containing 1 mg Cd/kg led to a decline (by 17–40%) in the concentration of Mn in the duodenum, except for a lack of change after 10 months (Figure 3). The exposure to the 5 mg Cd/kg diet decreased (by 13%) the concentration of Mn in the duodenum after 3 months and increased after 10 and 24 months (by 25% and 39%, respectively) (Figure 3). The administration of AE during the moderate treatment with Cd partially or entirely prevented the impact of this xenobiotic on the concentration of Mn in the duodenum (Figure 3).

The concentration of Mn in the stomach in the Cd5 group after 3 months was lower (by 9%) than in the Cd1 group, while the gastric and duodenal concentration of this element after 10 and 24 months was higher (by 23% to 2.3-fold) under the moderate exposure (Figure 3). The concentration of Mn in the stomach in the Cd5 + AE group after 10 months was lower (by 15%), while after 24 months it was higher (by 30%) than in the Cd1 + AE group (Figure 3). Moreover, the duodenal concentration of this element in the Cd5 + AE group after 3 and 24 months was higher (by 28% and 48%, respectively) compared to the Cd1 + AE group (Figure 3).

#### 3.2.3. The Concentration of Mn in the Serum and Tissues

**Figure 4:**
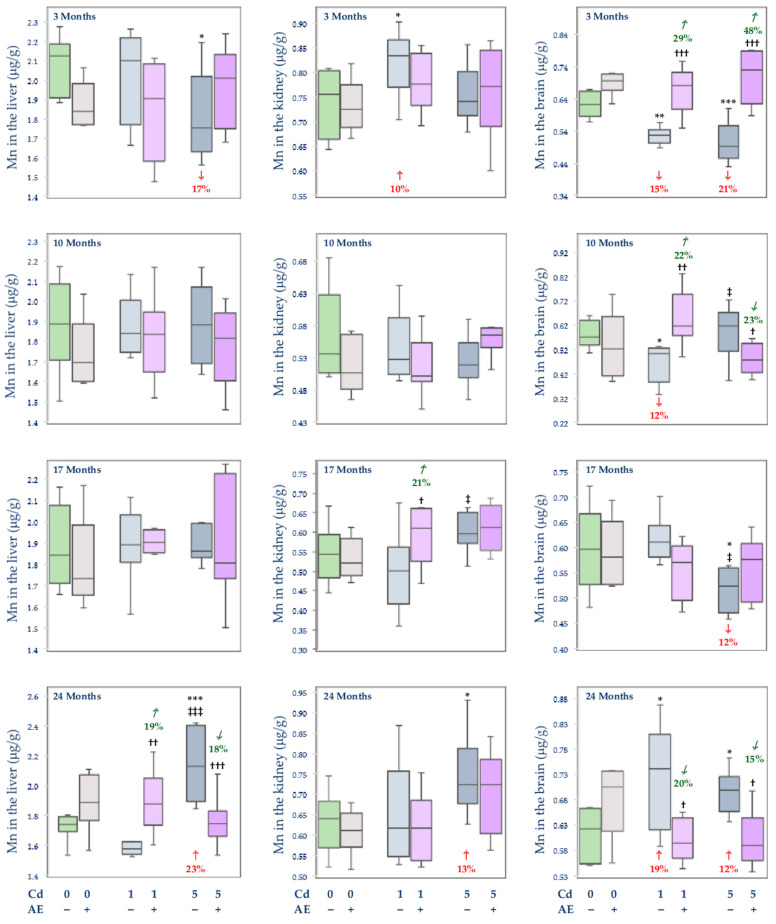
The concentration of Mn in the liver, kidney, and brain in particular experimental groups. The rats received Cd in the diet at the concentration of 0, 1, and 5 mg/kg and/or 0.1% AE as the only drinking fluid (“+”, administered; “−”, without administration). Data represent a median and 25–75% confidence interval (the horizontal lines within the bars presenting the confidence interval represent median values), as well as the minimum and maximum values for eight rats in each experimental group (except for seven animals in the AE, Cd1, and Cd5 groups after 24 months). Statistically significant differences: * *p* < 0.05, ** *p* < 0.01, *** *p* < 0.001 vs. control group; ^†^ *p* < 0.05, ^††^ *p* < 0.01, ^†††^ *p* < 0.001 vs. respective group receiving Cd alone (Cd1 or Cd5 group); ^‡^ *p* < 0.05, ^‡‡‡^ *p* < 0.001 vs. respective group receiving the 1 mg Cd/kg diet alone (Cd1 group) or with the AE (Cd1 + AE group) are marked. The values below and above the bars indicate percentage changes compared to the control group (↓, decrease; ↑, increase) or the respective group receiving Cd alone (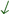, decrease; 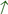, increase), respectively.

**Figure 5:**
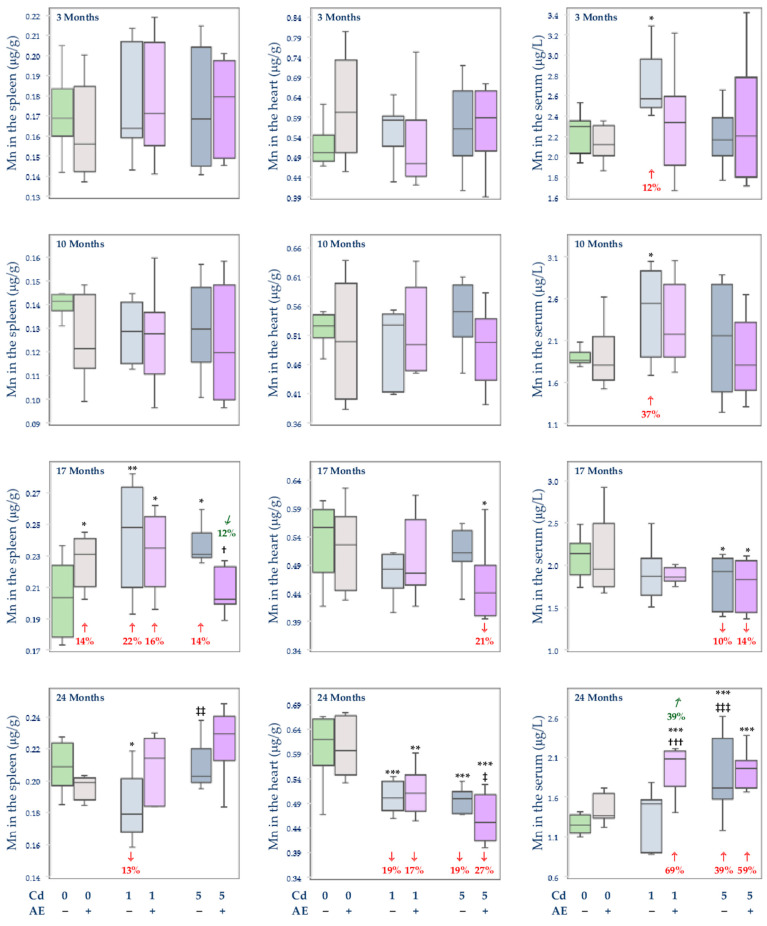
The concentration of Mn in the spleen, heart, and serum in particular experimental groups. The rats received Cd in the diet at the concentration of 0, 1, and 5 mg/kg and/or 0.1% AE as the only drinking fluid (“+”, administered; “−”, without administration). Data represent a median and 25–75% confidence interval (the horizontal lines within the bars presenting the confidence interval represent median values), as well as the minimum and maximum values for eight rats in each experimental group (except for seven animals in the AE, Cd1, and Cd5 groups after 24 months). Statistically significant differences: * *p* < 0.05, ** *p* < 0.01, *** *p* < 0.001 vs. control group; ^†^ *p* < 0.05, ^†††^ *p* < 0.001 vs. respective group receiving Cd alone (Cd1 or Cd5 group); ^‡^ *p* < 0.05, ^‡‡^ *p* < 0.01, ^‡‡‡^ *p* < 0.001 vs. respective group receiving the 1 mg Cd/kg diet alone (Cd1 group) or with the AE (Cd1 + AE group) are marked. The values below and above the bars indicate percentage changes compared to the control group (↓, decrease; ↑, increase) or the respective group receiving Cd alone (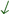, decrease; 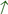, increase), respectively.

**Figure 6:**
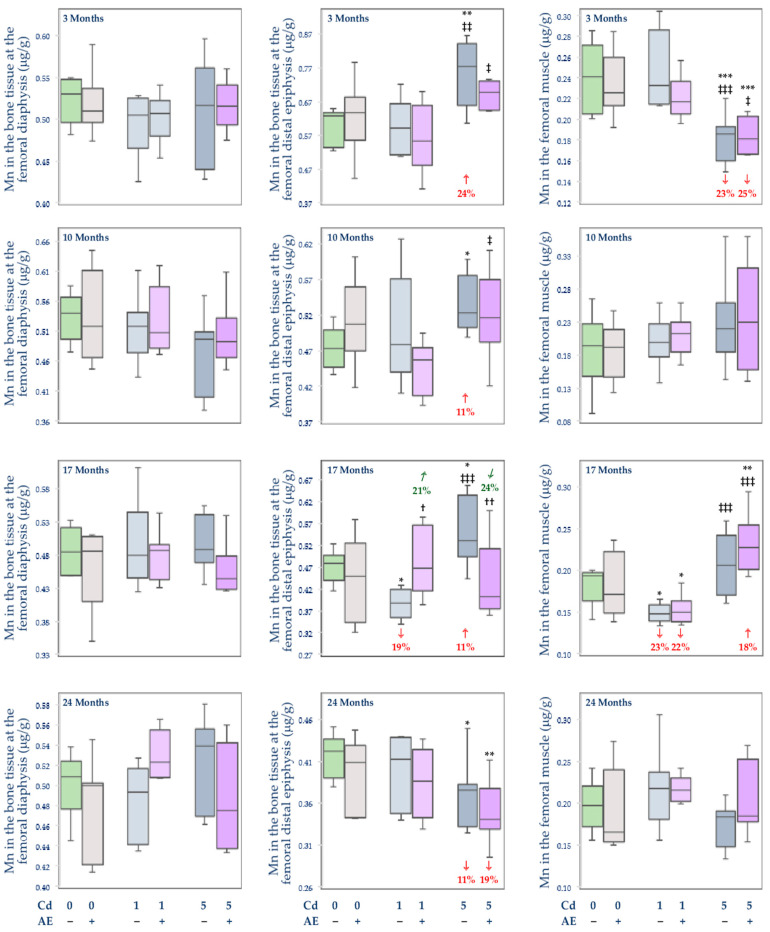
The concentration of Mn in the bone tissue and femoral muscle in particular experimental groups. The rats received Cd in the diet at the concentration of 0, 1, and 5 mg/kg and/or 0.1% AE as the only drinking fluid (“+”, administered; “−”, without administration). Data represent a median and 25–75% confidence interval (the horizontal lines within the bars presenting the confidence interval represent median values), as well as the minimum and maximum values for eight rats in each experimental group (except for seven animals in the AE, Cd1, and Cd5 groups after 24 months). Statistically significant differences: * *p* < 0.05, ** *p* < 0.01, *** *p* < 0.001 vs. control group; ^†^ *p* < 0.05, ^††^ *p* < 0.01, vs. respective group receiving Cd alone (Cd1 or Cd5 group); ^‡^ *p* < 0.05, ^‡‡^ *p* < 0.01, ^‡‡‡^ *p* < 0.001 vs. respective group receiving the 1 mg Cd/kg diet alone (Cd1 group) or with the AE (Cd1 + AE group) are marked. The values below and above the bars indicate percentage changes compared to the control group (↓, decrease; ↑, increase) or the respective group receiving Cd alone (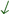, decrease; 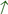, increase), respectively.

The administration of AE alone did not influence the concentration of Mn in the serum, soft tissues (liver, kidney, brain, spleen, heart, and femoral muscle), and bone tissue at the distal epiphysis and diaphysis of the femur (Figure 4, Figure 5 and Figure 6), apart from an elevation (by 14%) in this element concentration in the spleen after 17 months of the experiment (Figure 5).

Depending on the level and duration of intoxication, Cd variously influenced (increased or decreased) the concentration of Mn in the serum and investigated tissues (Figure 4, Figure 5 and Figure 6). The exposure to the 1 mg Cd/kg diet only at some time points influenced the concentrations of Mn in the serum (an increase after 3 and 10 months), kidney (an increase after 3 months), spleen (an increase after 17 months and decrease after 24 months), heart (a decrease after 24 months), bone tissue at the femoral distal epiphysis (a decrease after 17 months), and femoral muscle (a decrease after 17 months) (Figure 4, Figure 5 and Figure 6). However, almost throughout the whole experiment, the low-level treatment with Cd affected the brain concentration of this element leading to its decline after 3 and 10 months (by 15% and 12%, respectively) and elevation (by 19%) after 24 months (Figure 4). The co-treatment with the extract totally protected against the 1 mg Cd/kg diet-induced alterations in the concentration of Mn in the serum, kidney, brain, and bone tissue at the femoral distal epiphysis (Figure 4, Figure 5 and Figure 6). Moreover, the extract co-administration protected from the decrease in the concentration of Mn in the spleen after 24 months but not from its increase after 17 months (Figure 5). The extract administration did not modify the impact of Cd on the heart and femoral muscle concentration of this element and increased (by 39%) the Cd alone unchanged concentration of Mn in the serum after 24 months (Figure 5 and Figure 6).

In the group of rats fed with the 5 mg Cd/kg diet alone, an increase (by 11–24%) in the concentration of Mn in the bone tissue at the femoral distal epiphysis during the first 17 months and its decrease (by 11%) after 24 months, as well as a decrease in the concentration of this element in the brain after 3 and 17 months (by 21% and 12%, respectively) and its increase (by 12%) after 24 months (Figure 4 and Figure 6), were noted. The treatment with the 5 mg Cd/kg diet variously influenced also the concentration of Mn in the liver (a decrease by 17% after 3 months and an increase by 23% after 24 months; Figure 4) and serum (a decrease by 10% after 17 months and an increase by 39% after 24 months; Figure 5). Moreover, the concentration of Mn was decreased after 3 months of the treatment with the 5 mg Cd/kg diet in the femoral muscle (by 23%) and after 24 months in the kidney (by 13%) and heart (by 19%), while in the spleen it was increased (by 14%) after 17 months (Figure 4, Figure 5 and Figure 6). The administration of AE under the intoxication with the 5 mg Cd/kg diet prevented all Cd-caused changes in the concentration of Mn in the liver, kidney, brain, spleen, and bone tissue at the femoral distal epiphysis, except for the decrease in the bone tissue after 24 months (Figure 4, Figure 5 and Figure 6). However, the extract did not protect from the impact of the 5 mg Cd/kg diet on the concentration of Mn in the heart, serum, femoral muscle, and bone tissue at the femoral distal epiphysis (Figure 5 and Figure 6). Moreover, the intake of AE resulted in a decrease (by 21%) in the unaffected by Cd alone concentration of Mn in the heart and an increase (by 18%) in the concentration of this essential element in the femoral muscle after 17 months (Figure 5 and Figure 6).

In the animals fed with the diet containing 5 mg Cd/kg, at some time points, the concentration of Mn in the liver (24 months), kidney (17 months), brain (10 months), spleen (24 months), serum (24 months), distal femoral epiphysis (3 and 17 months), and femoral muscle (17 months) was higher (by 9–39%) than in those fed with the 1 mg Cd/kg diet, whereas this element concentration in the brain after 17 months and femoral muscle after 3 months was lower (by 12% and 20%, respectively) at the moderate exposure (Figure 4, Figure 5 and Figure 6). Moreover, the concentration of Mn in the Cd5 + AE group in the heart after 24 months and femoral muscle after 3 months was lower (by 12% and 17%, respectively), while in the distal femoral epiphysis after 3 and 10 months and in the femoral muscle after 17 months was higher (by 13–52%) compared to the Cd1 + AE group (Figure 5 and Figure 6).

#### 3.2.4. Total Content of Mn in Internal Organs

**Figure 7:**
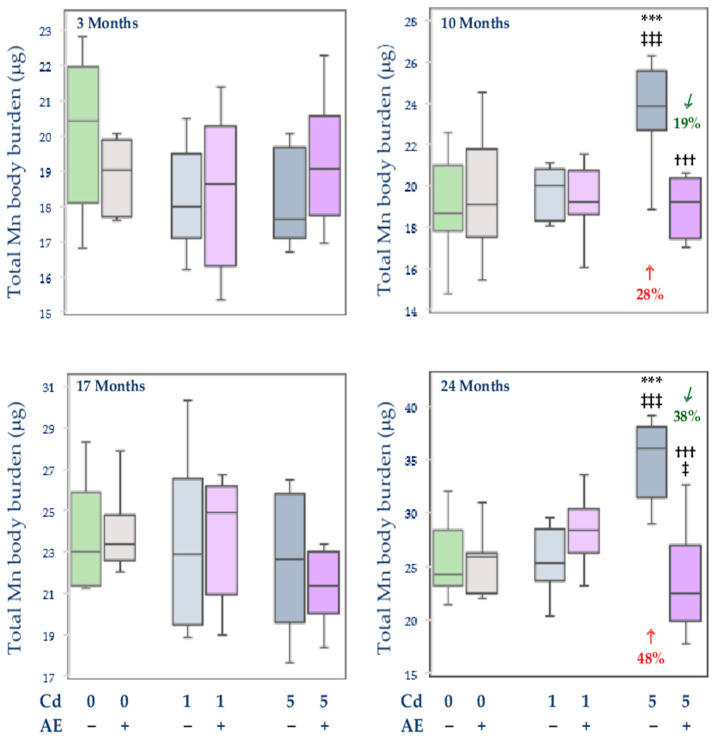
The total body burden of Mn in particular experimental groups. The rats received Cd in the diet at the concentration of 0, 1, and 5 mg/kg and/or 0.1% AE as the only drinking fluid (“+”, administered; “−”, without administration). Data represent a median and 25–75% confidence interval (the horizontal lines within the bars presenting the confidence interval represent median values), as well as the minimum and maximum values for eight rats in each experimental group (except for seven animals in the AE, Cd1, and Cd5 groups after 24 months). Statistically significant differences: *** *p* < 0.001 vs. control group; ^†††^ *p* < 0.001 vs. the Cd5 group; ^‡^ *p* < 0.05, ^‡‡‡^ *p* < 0.001 vs. respective group receiving the 1 mg Cd/kg diet alone (Cd1 group) or with the AE (Cd1 + AE group) are marked. The values below and above the bars indicate percentage changes compared to the control group (↑, increase) or the respective group receiving Cd alone (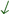, decrease), respectively.

There were no changes in the total body burden of Mn and its content in particular internal organs in the group administered with AE alone, except for an increase in the content of this element in the brain after 3 months (by 10%) and an increase in the spleen after 17 months of the investigation (by 29%) (Figure 7 and Table 3, Table 4, and Table S5).

The feeding with the diet containing 1 mg Cd/kg alone influenced the content of Mn only in the spleen (an increase by 23% after 3 months) and brain (a decrease by 12% after 3 and 10 months and an increase by 20% after 24 months) (Table 3 and Table 4). Nevertheless, the low intoxication with Cd did not influence the total content of Mn in the internal organs at any time point (Figure 7 and Table S5).

The exposure to the 5 mg Cd/kg diet alone resulted in an increase in the content of Mn in the spleen (by 21% after 3 months), liver (by 32% and 45% after 10 and 24 months, respectively), and kidney (by 27% after 24 months). The brain content of Mn decreased after 3 and 17 months (by 21% and 18%, respectively), while its content in the heart increased (by 22%) after 10 months and decreased (by 22%) after 24 months (Table 3 and Table 4). The 10- and 24-month feeding with the diet containing 5 mg Cd/kg led to an increase in the total body burden of Mn (by 28% and 48%, respectively) (Figure 7).

The treatment with the 5 mg Cd/kg diet caused more intense alterations in the content of Mn in the investigated organs compared to the 1 mg Cd/kg diet (Figure 7, Table 3, Table 4, and Table S5). In the Cd5 group, the content of Mn at some time points was higher than in the Cd1 group–in the liver (by 20% and 41% after 10 and 24 months, respectively), kidney (by 34% after 24 months), and brain (by 30% after 10 months). The brain content of this element at moderate exposure to Cd after 17 months was lower (by 14%) than at the low-level treatment. The total content of Mn in internal organs after 10 and 24 months in the Cd5 group was higher (by 19% and 42%, respectively) than in the Cd1 group (Figure 7 and Table S5).

The co-administration of the extract provided complete or partial protection against Cd-mediated changes in the content of Mn in particular organs, apart from the spleen (Table 3 and Table 4). Moreover, in the heart, the content of Mn after 24 months in the Cd5 + AE group was more markedly decreased compared to the control than in the Cd5 group (Table 4). The 3-month administration of AE to the animals maintained on the diet containing 5 mg Cd/kg not only protected from the Cd-induced decrease in the content of Mn in the brain but it increased the value of this parameter by 61% making it higher (by 28%) compared to the control group (Table 3). The application of AE under the low-level exposure to Cd did not influence the total content of Mn in internal organs, whereas its administration during the exposure to the 5 mg Cd/kg diet completely protected against this heavy metal-induced increase in the total body burden of Mn after 10 and 24 months (Figure 7).

In the animals co-administered with the extract and the 5 mg Cd/kg diet, the content of Mn at some time points was lower in the spleen (by 17% after 17 months), brain (by 24% after 10 months), and heart (by 18% after 24 months) compared to the Cd1 + AE group, whereas in the brain it was higher (by 22%) after 3 months (Table 3 and Table 4). There were no differences in the content of Mn between the groups co-administered with this toxic metal and AE, except for a lower (by 21%) total body burden of Mn in the Cd5 + AE group compared to the Cd1 + AE group after 24 months (Figure 7).

### 3.3. Effect of Cd and/or AE on the Activity of MnSOD, and the Concentrations of Mn and Cd in the Mitochondrial Fraction of the Liver, Kidney, and Brain

**Figure 8:**
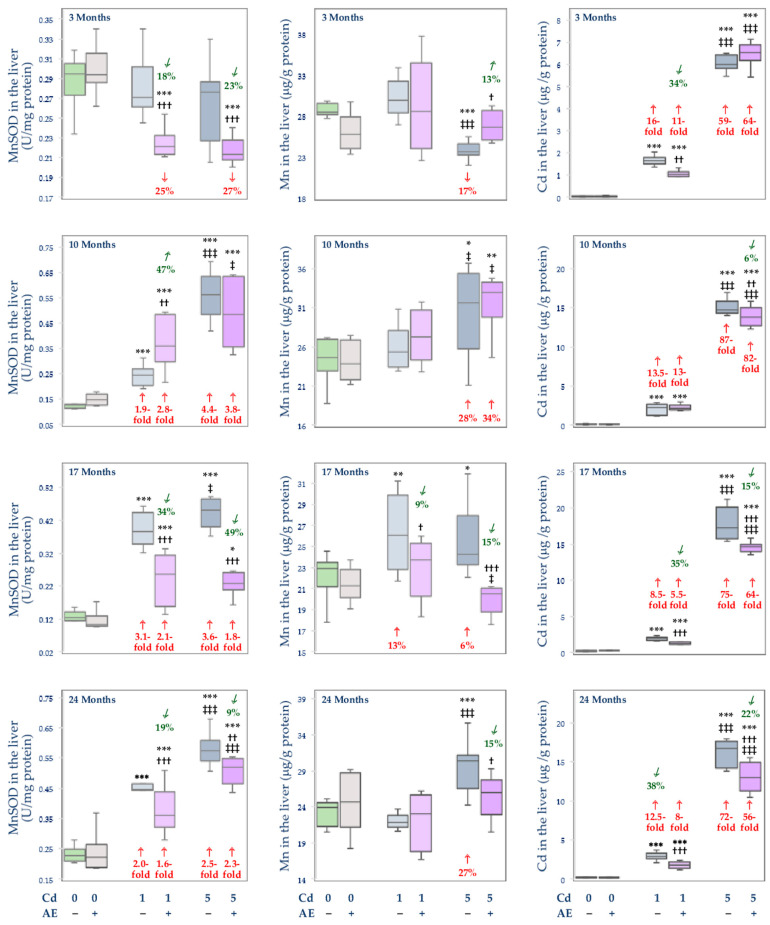
The activity of MnSOD and the concentrations of Mn and Cd in the mitochondrial fraction of the liver in particular experimental groups. The rats received Cd in the diet at the concentration of 0, 1, and 5 mg/kg and/or 0.1% AE as the only drinking fluid (“+”, administered; “−”, without administration). Data represent a median and 25–75% confidence interval (the horizontal lines within the bars presenting the confidence interval represent median values), as well as the minimum and maximum values for eight rats in each experimental group (except for seven animals in the AE, Cd1, and Cd5 groups after 24 months). Statistically significant differences: * *p* < 0.05, ** *p* < 0.01, *** *p* < 0.001 vs. control group; ^†^ *p* < 0.05, ^††^ *p* < 0.01, ^†††^ *p* < 0.001 vs. respective group receiving Cd alone (Cd1 or Cd5 group); ^‡^ *p* < 0.05, ^‡‡‡^ *p* < 0.001 vs. respective group receiving the 1 mg Cd/kg diet alone (Cd1 group) or with the AE (Cd1 + AE group) are marked. The values below and above the bars indicate percentage changes or folds of changes compared to the control group (↓, decrease; ↑, increase) or the respective group receiving Cd alone (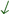, decrease; 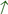, increase).

**Figure 9:**
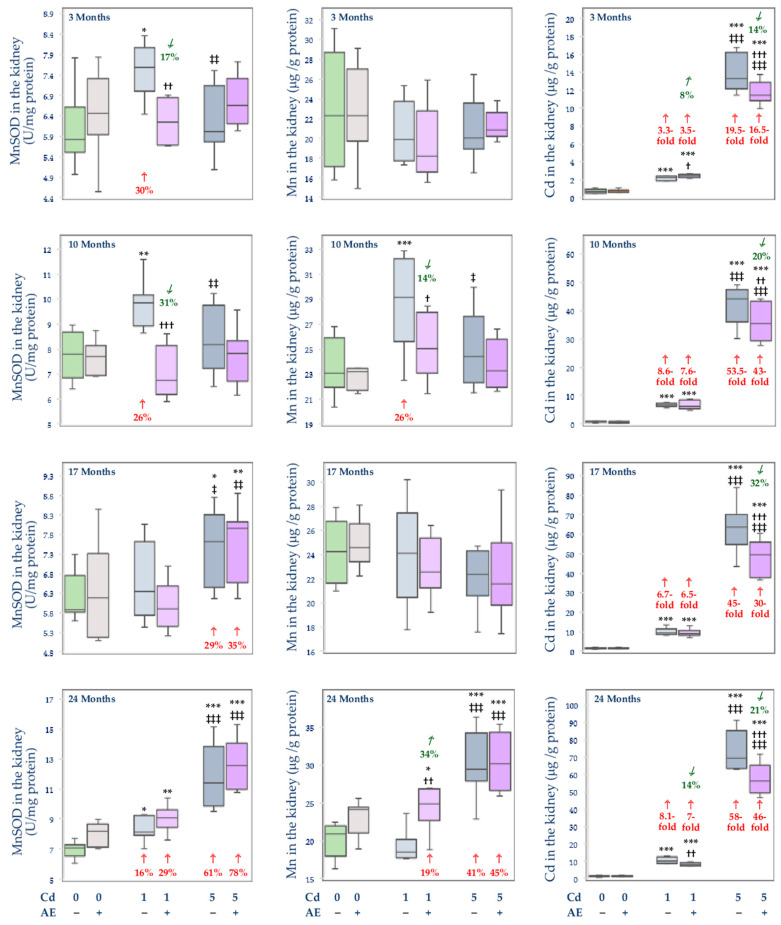
The activity of MnSOD and the concentrations of Mn and Cd in the mitochondrial fraction of the kidney in particular experimental groups. The rats received Cd in the diet at the concentration of 0, 1, and 5 mg/kg and/or 0.1% AE as the only drinking fluid (“+”, administered; “−”, without administration). Data represent a median and 25–75% confidence interval (the horizontal lines within the bars presenting the confidence interval represent median values), as well as the minimum and maximum values for eight rats in each experimental group (except for seven animals in the AE, Cd1, and Cd5 groups after 24 months). Statistically significant differences (Kruskal-Wallis post hoc test): * *p* < 0.05, ** *p* < 0.01, *** *p* < 0.001 vs. control group; ^†^ *p* < 0.05, ^††^ *p* < 0.01, ^†††^ *p* < 0.001 vs. respective group receiving Cd alone (Cd1 or Cd5 group); ^‡^ *p* < 0.05, ^‡‡^ *p* < 0.01, ^‡‡‡^ *p* < 0.001 vs. respective group receiving the 1 mg Cd/kg diet alone (Cd1 group) or with the AE (Cd1 + AE group) are marked. The values below and above the bars (or above the bars) indicate percentage changes or folds of changes compared to the control group (↑, increase) or the respective group receiving Cd alone (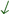, decrease; 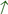, increase).

**Figure 10:**
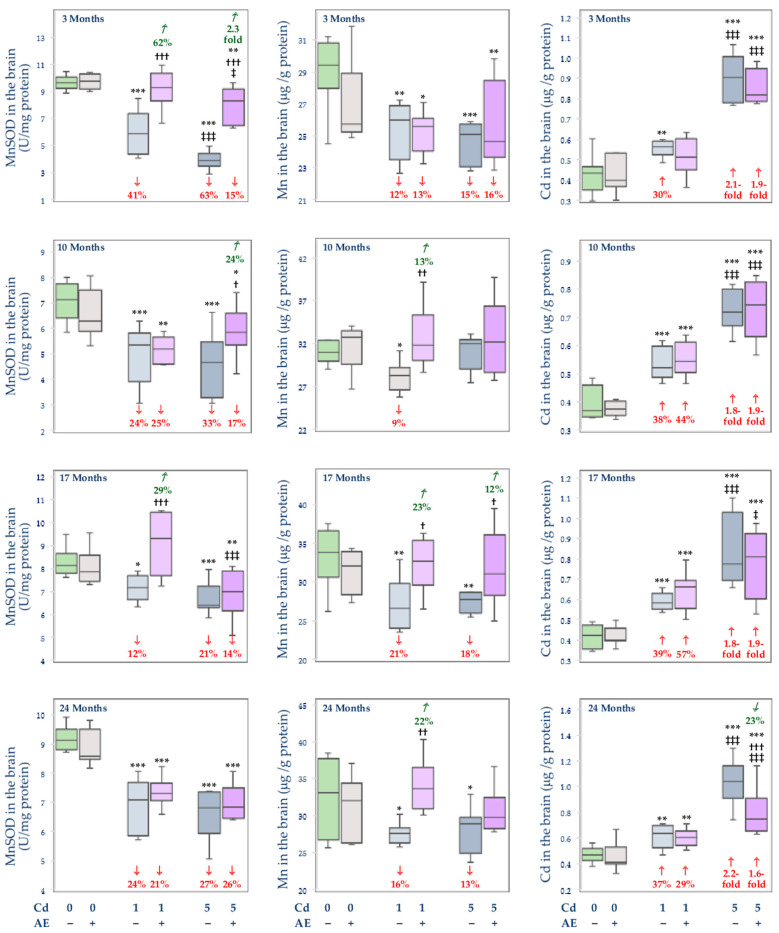
The activity of MnSOD and the concentrations of Mn and Cd in the mitochondrial fraction of the brain in particular experimental groups. The rats received Cd in the diet at the concentration of 0, 1, and 5 mg/kg and/or 0.1% AE as the only drinking fluid (“+”, administered; “−”, without administration). Data represent a median and 25–75% confidence interval (the horizontal lines within the bars presenting the confidence interval represent median values), as well as the minimum and maximum values for eight rats in each experimental group (except for seven animals in the AE, Cd1, and Cd5 groups after 24 months). Statistically significant differences: * *p* < 0.05, ** *p* < 0.01, *** *p* < 0.001 vs. control group; ^†^ *p* < 0.05, ^††^ *p* < 0.01, ^†††^ *p* < 0.001 vs. respective group receiving Cd alone (Cd1 or Cd5 group); ^‡^ *p* < 0.05, ^‡‡‡^ *p* < 0.001 vs. respective group receiving the 1 mg Cd/kg diet alone (Cd1 group) or with the AE (Cd1 + AE group) are marked. The values below and above the bars indicate percentages change or folds of changes compared to the control group (↓, decrease; ↑, increase) or the respective group receiving Cd alone (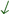, decrease; 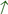, increase), respectively.

The administration of AE alone had no impact on the activity of MnSOD and the concentrations of Mn and Cd in the mitochondrial fraction of the liver, kidney, and brain (Figure 8, Figure 9 and Figure 10).

Generally, the exposure to Cd led to an increase in the activity of MnSOD in the mitochondrial fraction of the liver and kidney (from 16% to 4.4-fold) (Figure 8 and Figure 9) and a decrease in its activity in the brain (by 12–63%) (Figure 10). The co-treatment with the extract provided partial protection against this xenobiotic-caused increase in this parameter in the liver after 17 and 24 months (Figure 8) and prevented its change in the kidney, but only after 3 and 10 months of the study (Figure 9). Moreover, the administration of AE totally or partially prevented Cd-induced disturbances in the activity of MnSOD in the brain for up to 17 months, apart from the Cd1 + AE group after 10 months and the Cd5 + AE group after 17 months (Figure 10). The 24-month co-administration of AE at both levels of exposure to Cd did not influence this heavy metal-enhanced activity of MnSOD in the brain (Figure 10).

The low-level and moderate intoxication with Cd resulted in an increase in the concentration of Mn in the mitochondria of the liver (by 6–28%) and kidney (by 26–41%) at some time points, except for the Cd5 group after 3 months in which the concentration of Mn in the liver was lower (by 17%) than in the control group (Figure 8 and Figure 9). The co-administration of AE prevented the Cd-induced alterations in the concentration of Mn in the mitochondria of the liver during the 3-, 17-, and 24-month study (but not after 10 months) and of the kidney after 10 months (Figure 8 and Figure 9). The extract administration under the treatment with the 5 mg Cd/kg diet did not protect from the increase in the concentration of Mn in the mitochondria of the liver after 10 months and its concentration in the mitochondria of the kidney after 24 months. Moreover, the administration of AE during the feeding with the 1 mg Cd/kg diet increased the Cd-unchanged concentration of Mn in the mitochondria of the kidney after 24 months making it higher by 19% than in the control group (Figure 9). The concentration of Mn in the mitochondrial fraction of the brain was decreased (by 9–21%) at both levels of exposure to Cd, except for the 10-month intoxication with the 5 mg Cd/kg diet (Figure 10). The extract co-administration for 10–24 months prevented the Cd-induced drop in the concentration of Mn, whereas its application for 3 months had no impact on this bioelement concentration in the mitochondria of the brain under the low-level and moderate treatment with Cd.

The concentration of Cd in the mitochondria of rats maintained on the diet containing 1 and 5 mg Cd/kg was elevated in the liver (8.5–87-fold), kidney (3.3–58-fold), and brain (by 30% to 2.2-fold) (Figure 8, Figure 9 and Figure 10). The extract administration under the low-level and moderate exposure to Cd partially protected from this toxic metal accumulation in the liver mitochondria except for the Cd1 + AE group after 10 months and the Cd5 + AE group after 3 months. At all other time points at both levels of exposure to Cd and AE co-administration, the concentration of this heavy metal in the mitochondria was lower by 6–38% compared to the respective groups treated with Cd alone. The extract partially protected against the increase in the concentration of Cd in the mitochondria of the kidney under the whole exposure to the 5 mg Cd/kg diet and during the 24-month treatment with the 1 mg Cd/kg diet (Figure 9). However, the 3-month co-administration of AE caused an increase (by 8%) in the value of this parameter in the kidney compared to the Cd1 group and its 17-month co-administration had no impact (Figure 9). Moreover, the co-administration of AE did not modify Cd accumulation in the mitochondria of the brain apart from the Cd1 + AE group after 3 months (complete protection) and Cd5 + AE group after 24 months (partial protection) (Figure 10).

The activity of MnSOD in the mitochondria of the liver (10–24 months) and kidney (17 and 24 months) in the Cd5 group was higher (from 17% to 1.3-fold) than in the Cd1 group in the liver and was lower in the brain (by 38% after 3 months) and kidney (by 21% and 17% after 3 and 10 months, respectively) (Figure 8, Figure 9 and Figure 10). Moreover, the activity of this enzyme in the mitochondria in the Cd5 + AE group was higher (by 17–44%) in the liver (10 and 24 months) and kidney (17 and 24 months) and lower in the brain (by 11% and 24% after 3 and 17 months, respectively) compared to the Cd1 + AE group (Figure 4, Figure 5 and Figure 6). In the Cd5 group, the concentration of Mn in the mitochondria of the liver was lower (by 21%) after 3 months and higher after 10 and 24 months (by 25% and 39%, respectively) compared to the Cd1 group, whereas in the Cd5 + AE group the value of this parameter was higher (by 21%) after 10 and lower (by 14%) after 17 months compared to the Cd1 + AE group (Figure 8). The concentration of Mn in the mitochondria did not differ depending on the level of exposure to Cd in the brain but in the kidney, it was lower (by 16%) in the Cd5 group compared to the Cd1 group after 10 months and higher (by 59%) after 24 months (Figure 9 and Figure 10). Moreover, the concentration of Mn in the Cd5 + AE group was higher (by 21%) than in the Cd1 + AE group. The concentration of Cd in the mitochondria in all investigated tissues was higher under moderate exposure to this toxic heavy metal with or without AE co-administration at every time point (from 23% to 11-fold) (Figure 8, Figure 9 and Figure 10).

The activity of MnSOD in the mitochondria of the liver and kidney positively correlated with the concentrations of Mn (*β* = 0.323, R^2^ = 0.104, *p* < 0.01 and *β* = 0.461, R^2^ = 0.212, *p* < 0.001, respectively) and Cd (*β* = 0.758, R^2^ = 0.575, *p* < 0.001 and *β* = 0.500, R^2^ = 0.250, *p* < 0.01, respectively). This enzyme activity in the mitochondria of the brain also positively correlated with the concentration of Mn (*β* = 0.352, R^2^ = 0.124, *p* < 0.001) but negatively with the concentration of Cd (*β* = −0.564, R^2^ = 0.318, *p* < 0.001).

## 4. Discussion

The present study is the first investigation showing that low-level and moderate repeated exposure to Cd may affect the body burden of Mn and the activity of MnSOD in the mitochondria and that the co-administration of an extract from the berries of *A. melanocarpa* protects against these effects. The importance of the findings is enhanced by performing the experiment in a rat model well reflecting environmental human exposure to Cd in industrialized countries. These findings allow concluding that even low-level chronic exposure to Cd may pose a danger of disturbing the homeostasis of Mn in the human body, and the antioxidative function of the mitochondria, while the consumption of products based on aronia berries may ameliorate the impact of this heavy metal.

In studies investigating the possible protective effect of one factor against the toxic action of the other factor, it is very important to ensure the same intake of both factors, whether they are administered together or separately. Thus, during the study, the daily consumptions of the diet and 0.1% AE in particular experimental groups were monitored. The finding that the daily intake of Mn from all sources did not differ, regardless of whether the extract was administered alone or together with Cd, confirmed the appropriateness of the used experimental model to investigate the impact of these two factors co-administration on the body status of this essential element. Due to the very low concentration of Mn in the 0.1% AE, the daily intake of this bioelement via the extract was negligible compared to its intake with the Labofeed diets and thus the daily intake of this element was evaluated based on its intake via the diet. Because the daily intake of Mn during the first 3 months of the study (Labofeed H diet) was higher than thereafter (Labofeed B diet), its concentration in the serum and some tissues of the control animals was also somewhat higher than at the other time points. However, the content of this element in particular organs and its total body burden in the control animals were relatively stable throughout the 24-month study. Moreover, the intake of Mn at particular time points did not differ among all experimental groups, and thus the fact of the higher intake of this bioelement at the beginning of the study had no impact on the results of this experiment.

The present investigation showed that Cd can disturb the homeostasis of Mn in the organism and that this effect is different depending on the level and duration of the intoxication and the type of the organ or tissue. The exposure to the 1 and 5 mg Cd/kg diet altered the turnover of Mn already at the stage of its intestinal absorption, as well as influenced its retention in the body and the excretion with faeces and urine. As a result, the concentration and/or content of Mn at various time points in most of the investigated tissues, except for its concentration in the bone tissue at the femoral diaphysis, were modified. The Cd-mediated disturbances in the concentration of Mn might stem from the competition between these metals for the same transport systems [29,44]. It has been shown that both Cd and Mn have an affinity for Zn transporters, such as Zrt, Irt-related protein 8 (ZIP8) and ZIP14, and divalent metal transporter 1 (DMT1) [35]. Therefore, it is reasonable to assume that disruption of Mn homeostasis, in part, especially a decrease in this element concentration in the stomach and duodenum, reflects changes in its gastrointestinal absorption, which can occur during Cd intoxication. The unchanged Mn concentration in the bone tissue at the femoral diaphysis despite the changes in its concentration in other tissues, including the distal epiphysis of the femur, may be explained by the fact that in this region of the femur the less metabolically active and thus less vulnerable to be destroyed cortical bone predominates. The decrease in the concentration of Mn in the bone tissue at the femoral distal epiphysis in the Cd5 group after 24 months may be explained by enhanced bone resorption previously reported in these animals [32]. However, taking into account the enhanced bone resorption, it is difficult to explain why the concentration of Mn in this bone region was enhanced after 3, 10, and 17 months. In the present research, based on the concentration of Mn in the internal organs of the rats exposed to Cd, it is hard to clearly define the main direction of changes in this parameter. However, taking into account that the content of any bioelement in particular organs and its total content in these organs may be better measures to reflect the body status of this element than its concentration, we may assume that the Cd-mediated alterations in the turnover of Mn consisted in an increase in the content of this necessary element in internal organs creating the potential risk of harmful outcomes.

Recently, more attention has been paid to the involvement of metals, including both Cd and Mn, in the pathogenesis of neurodegenerative diseases [6,21,45,46]. The results of the present study show that both low-level and moderate long-term exposure to Cd may result in the retention of Mn in the brain. Although the brain content of Mn in the Cd5 group after 24 months did not differ compared to the control group, however, it reached the median value clearly higher than in the control group and the concentration of this element in the brain at this time point was increased. Moreover, the decreased concentration of Mn in the mitochondrial fraction of the brain after 24 months, despite its enhanced concentration in the brain tissue, shows that Cd caused a redistribution of this bioelement in the brain. Elevated levels of Mn in the brain are usually an outcome of increased exposure to this element; therefore, showing a growth in the cerebral concentration of Mn as a consequence of the low-level and moderate lifetime (24 months in the used experimental model) exposure to Cd is an important issue concerning the toxicity of both elements, especially regarding the fact that the brain is the target organ for the toxic action of Mn^2+^ [4]. Although Mn is recognized to accumulate in the brain [4], the subcellular and organelle distribution of this element and the consequences of its disturbance are still far from being fully elucidated. Mn is known to accumulate in the mitochondria of neurons; however, the latest study by Carmona et al. [47] showed that in the dopaminergic cells, Mn predominantly accumulates in the Golgi apparatus and when the storage capacity in these organelles is exceeded this element is accumulated in the nucleus and cytoplasm. Therefore, the observed increase in the concentration of Mn in the brain in the present study and the decrease in its concentration in the mitochondria may be a result of an elevated deposition of this biometal in other cellular organelles, as well as in the cytoplasm.

The literature data indicate that Cd may variously influence the concentration of Mn in the internal organs or have no impact on it [9,10,12,29,30,48]. The differences in Cd-mediated changes in the distribution of Mn in particular organs and time points may be related to some differences in the induction of MT [10]. Eybl and Kotzova [10] reported that the administration of manganese chloride (20 mg MnCl_2_/kg b.w.) before intoxication with Cd (7 mg CdCl_2_/kg b.w.) significantly increased the accumulation of this toxic metal in the liver with its concomitant decrease in the kidneys and testis suggesting that this effect was a result of a greater ability of the liver to induce the biosynthesis of MT. The study by Goering and Klaassen [30] indicated that pre-treatment with Mn^2+^ (250 μmoles/kg b.w., subcutaneously–*s.c.*) altered the hepatic distribution of Cd (31 μmoles Cd^2+^/kg b.w., intravenously) in the cell with more Cd^2+^ bound to MT in the cytosol. The study showed that the hepatic concentration of Mn was increased in mice co-treated with Cd and Mn compared to the ones administered with this bioelement alone [30].

The positive relationships noted between the concentration of Mn and the activity of MnSOD in the mitochondria of the liver, kidney, and brain allow for the conclusion that the Cd-induced changes in the homeostasis of Mn in the mitochondria are related to changes in the antioxidative capacity of these organelles. Oxidative stress has been recognized as the major mechanism of the damaging action of Cd and mitochondria are considered the target cellular organs for the toxic action of this xenobiotic [9,10,12,15,20,22,23,24,25,34,37,49,50,51,52]. Several studies have reported that Cd may induce apoptosis through the breakdown of mitochondrial membrane potential and overproduction of ROS [15,37,51,52]. A decrease in the activity of MnSOD, catalyzing the reaction of dismutation of O_2_^−^ to hydrogen peroxide (H_2_O_2_) and molecular oxygen [11,12,13,14], may increase the concentration of O_2_^−^ in the mitochondria. On the other hand, its elevation may lead to the increased production of H_2_O_2_ which, if not efficiently detoxified, may be the source of extremely reactive **^·^**OH [13,14]. Therefore, the proper activity of this enzyme is necessary to maintain the oxidative/reductive balance in these organelles. Moreover, it has been shown that the mitochondrial isoform of SOD is more susceptible to the impact of Cd than its cytosolic isoform (CuZnSOD) and catalase (CAT) [12]. The disruption of the concentration of Mn in the mitochondria of the liver, kidney, and brain noted in the present study due to the exposure to Cd was accompanied by changes in the activity of MnSOD in these organelles. In the liver and kidney, the Cd-induced increase in the concentration of Mn was associated with the increase in the activity of MnSOD, while in the brain, where the intoxication with this toxic metal caused a decrease in the concentration of Mn, the activity of this enzyme was also decreased. It can be surmised that the increase in the activity of MnSOD in the mitochondria of the liver and kidney was an effect of both the increase in the concentration of Mn in these structures and the result of the defense mechanisms and/or adaptive response of these organelles against Cd-mediated increase in ROS production caused by the mitochondrial accumulation of this xenobiotic. The decrease in the activity of MnSOD in the mitochondria in the brain might result from the decreased concentration of Mn and the substitution of this bioelement by Cd in the active center of this enzyme [12]. The decrease in the activity of MnSOD in the brain is especially disturbing regarding the literature reports indicating that this enzyme protects the neural cells from apoptosis and a decrease in its activity is associated with neurodegeneration [53,54].

The most important achievement of the present work is the finding that the administration of the 0.1% AE diminished the Cd-caused disturbance of the body status of Mn and this bioelement homeostasis in the mitochondria, as well as the activity of MnSOD in these organelles in the liver, kidney, and brain in the rats intoxicated with the 1 and 5 mg Cd/kg diet. Moreover, considering the metal-chelating properties of the extract ingredients [26,31,35,36], a very important outcome of this study is showing that the long-term consumption of AE alone does not create a danger of Mn deficiency in the organism. The literature data indicate a protective effect of low doses of Mn against Cd toxicity [9,10,11,12,16,30,44]; therefore, the maintenance of this bioelement homeostasis may support the protective action of AE during exposure to this heavy metal. Although the occurrence and the level of protection provided by AE were different at particular time points and levels of exposure to Cd, nevertheless, in general, the extract was effective in protecting from Cd-mediated disturbances in the concentration and content of Mn in the internal organs, and most of all it provided complete protection from the effects caused by exposure to this toxic metal increase in the total body burden of Mn. The extract also prevented changes induced by Cd in the concentration of Mn in the mitochondria and mitigated the impact of this xenobiotic on the activity of MnSOD.

The effect of the supplementation with AE on the investigated parameters in the rats fed with the diet containing 1 and 5 mg Cd/kg might result from the independent impact of the extract, as well as from its interactive action with Cd. The independent action of AE might be a result of the high antioxidative potential of the extract conditioned by its ingredients, among other polyphenols, vitamins, β-carotene, and trace elements such as iron, selenium, and Zn [39,49,50]. From the prophylactic and therapeutic point of view, polyphenolic compounds are the most abundant and most important group of biologically active substances occurring in the berries of *A. melanocarpa* [32,39,49,50,55,56,57]. It has been shown that polyphenols and polyphenol-rich products support the antioxidative/oxidative balance of the cells including the prevention of Cd-induced disturbances in the activity of antioxidative enzymes [22,23,58,59]. In our previous investigation conducted in the model used in the present study, we noted that AE prevented changes in the activity of CuZnSOD in the liver and bone tissue of rats chronically exposed to Cd [22,23]. Mitra et al. [58] revealed the protective action of an extract from Curry leaf (*Murraya koenigii*; 100 mg/kg b.w., orally) against Cd-induced (0.44 mg CdCl_2_/kg b.w., *s.c.*) decrease in the activity of CuZnSOD and MnSOD in the cardiac tissue of rats. Elmallah et al. [59] reported that the extract from strawberry (*Fragaria ananassa*; 250 mg/kg b.w., orally) restored the activity of SOD up to the control values and enhanced the expression of MnSOD in the testis of rats intoxicated with Cd (6.5 mg CdCl_2_/kg b.w., intraperitoneally–*i.p.*). On the other hand, the study by Merra et al. [60] showed that hydroxytyrosol (9 mg/kg b.w., *i.p.*), a polyphenol most abundantly occurring in olive oil, prevented Cd-induced (2.5 mg CdCl_2_/kg b.w., *i.p.*) decrease in the activity of CuZnSOD but not MnSOD in the spleen and testis of mice. Taking into account our previous findings [22,23], it can be hypothesized that AE may protect against Cd-induced disturbances in the activity of both cytosolic and mitochondrial SOD. The interaction between AE and Cd is evoked by the metal-chelating abilities of the extract components, especially polyphenols [31,35]. The antagonistic nature of the simultaneous impact of Cd and AE on the investigated parameters may result from this extract-caused reduction of the concentration and content of this toxic element in the tissues [31]. Previously, we reported that the chokeberry extract decreased the intestinal absorption of Cd and its retention in the organism and, as a result, reduced the accumulation of this xenobiotic in the body [31]. Taking the above into consideration, it seems reasonable to assume that the effect of AE on the body status of Mn under exposure to Cd was determined by decreased gastrointestinal absorption of Cd and the lower amount of this xenobiotic available to penetrate the tissues and possibly lower expression of MT [24,31]. In turn, its favourable effect on the activity of MnSOD may stem from the strong antioxidative properties of AE and its ability to support the antioxidative/oxidative balance in the cells and prevent the induction of oxidative stress, as well as from the protection against Cd-mediated disturbances in the concentration of Mn in the mitochondria [12,22,23]. The positive correlations between the concentration of Mn and the activity of MnSOD in these organelles of the liver and kidney confirm this mechanism. However, it is unclear where the underlying bioinorganic processes that form the biomolecular basis for the obtained results unfold and that this could include the bloodstream or the organs themselves.

Among all of the achievements of the present investigation, there are also some limitations. The first is that the changes of the investigated parameters at particular time points were not studied in the same rats, but in subgroups of animals within the same experimental groups, which may explain why some effects of the exposure to Cd and/or administration of AE were observed or not at some time points. Moreover, considering that the investigation was performed on female rats, the results of this research primarily refer to females. Taking into account that females are more susceptible to Cd toxicity than males, it can be hypothesized that the toxic action of this xenobiotic on the body status of Mn and the mitochondrial activity of MnSOD will be less severe in males.

## 5. Conclusions

It has been shown, for the first time, that polyphenol-rich extract from the berries of *A. melanocarpa* protects against the destruction of the body status of Mn and the activity of MnSOD in the mitochondria due to chronic low and moderate exposure to Cd. Because it has been revealed in an animal model of human environmental exposure to Cd that even low-level lifetime treatment with this heavy metal may lead to the retention of Mn in the brain, which is a factor contributing to the development of neurodegenerative changes, this finding may throw new light on this xenobiotic neurotoxicity and the possibility of prevention from this action. The study confirms that both low and moderate treatment with this xenobiotic creates a risk of unfavourable consequences for the organism. Another valuable outcome of this investigation is showing that prolonged consumption of the chokeberry extract did not create a danger of disturbance in the homeostasis of Mn in the organism. The current study provided further evidence for the effectiveness of the extract in preventing the harmful consequences of exposure to Cd. Taking together the findings of the present investigation as well as the outcomes of our former research conducted in the same experimental model [22,23,24,34], it seems justifiable to pinpoint aronia berries products as possible candidates for effective prevention from the consequences for health resulting from exposure to this metal in humans. Thus, the findings have both scientific and practical values.

## Figures and Tables

**Table 1:** The ranges of the daily intakes of AE, polyphenols, and Cd during the 24-month experiment ^1^.

Group	Daily Intake during the 24-Month Administration ^1^	
	AE [Polyphenols] (mg/kg b.w.)	Cd (μg/kg b.w.)
Control	-	2.30–4.98
0.1% AE (AE)	67.4–146.6 [44.3–96.4]	2.25–4.95
1 mg Cd/kg (Cd1)	-	39.2–83.8
1 mg Cd/kg + 0.1% AE (Cd1 + AE)	67.2–154.7 [44.2–101.7]	37.5–84.9
5 mg Cd/kg (Cd5)	-	210.1–403.2
5 mg Cd/kg + 0.1% AE (Cd5 + AE)	63.1–150.3 [41.5–98.8]	200.2–401.9

^1^ Data represent the range of the daily intake of AE, polyphenols, or Cd throughout the 24-month study. Polyphenols intake was calculated assuming that the extract contained 65.74% of these compounds (certified value). Cd intake in the control animals and AE group was calculated based on its concentration in the standard diet (0.0584 ± 0.0049 mg/kg) [31], while Cd intake in the groups exposed to this metal was calculated based on its concentration in the diet as specified by the manufacturer (1 or 5 mg Cd/kg). Detailed data on the intakes of polyphenols and Cd in particular experimental groups during the 3, 10, 17, and 24 months of the study have already been published [31]. The intakes of AE and polyphenols in the control group and 1 and 5 mg Cd/kg groups were assumed to be 0.

**Table 2:** The intake of Mn with the Labofeed diets in particular experimental groups ^2^.

Group	Mn Intake (mg/kg b.w./24 h)			
	3 Months	10 Months	17 Months	24 Months
Control	11.479 10.830–12.038	5.347 *** 5.111–6.278	5.182 *** 4.523–5.409	5.652 *** 5.397–6.276
AE	11.545 10.748–12.294	5.805 *** 5.278–5.917	5.002 *** 4.619–5.408	5.641 *** 5.336–6.106
Cd1	11.565 10.804–12.320	5.591 *** 5.291–6.027	5.095 *** 4.762–5.429	5.744 *** 5.499–6.296
Cd1 + AE	11.538 10.983–11.900	5.587 *** 5.114–6.169	4.988 *** 4.762–5.655	5.895 *** 5.268–6.878
Cd5	11.141 10.679–11.740	5.620 *** 5.336–5.959	5.020 *** 4.542–5.211	5.853 *** 5.559–6.944
Cd5 + AE	11.335 10.785–11.655	5.720 *** 5.620–5.964	4.956 *** 4.743–5.289	5.870 *** 5.329–6.931

^2^ The intake of Mn was calculated based on its concentration in the Labofeed diets declared by the manufacturer in the certificate (145 mg Mn/kg in the Labofeed H diet administered throughout the first 3 months and 120 mg Mn/kg in the Labofeed B diet used thereafter). Due to the higher content of Mn in the Labofeed H diet than in the Labofeed B diet, the intake of this element during the first 3 months of the study was higher than during the 10, 17, and 24 months. Data represent a median value and minimum and maximum intake of Mn for 32, 24, 16, and 8 rats during 3, 10, 17, and 24 months, respectively, except for 7 animals in the AE, Cd1, and Cd5 groups in the last time-point of the experiment. *** *p* < 0.001 compared to the intake during the first 3 months.

**Table 3:** The total content of Mn in the internal organs in particular experimental groups after 3 and 10 months of the study ^3^.

Group	Mn Content (μg)				
	Liver	Kidneys	Spleen	Brain	Heart
3 Months					
Control	17.203 14.007–19.909	1.4302 1.1793–1.5344	0.1108 0.1030–0.1272	1.0769 0.9949–1.2456	0.4666 0.3760–0.5078
AE	15.641 14.325–16.757	1.4062 1.3373–1.5276	0.1124 0.0894–0.1448	1.1863 * 1.0755–1.3064 ↑ 10%	0.5008 0.3637–0.6853
Cd1	14.922 13.440–17.265	1.5174 1.2470–1.6783	0.1367 * 0.1113–0.1539 ↑ 23% ^4^	0.9457 ** 0.8961–0.9945 ↓ 12%	0.4612 0.3266–0.5053
Cd1 + AE	15.434 12.265–18.272	1.4570 1.3324–1.5486	0.1212 0.1005–0.1480	1.1308 ^†††^ 1.0169–1.3611 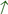 20%	0.4016 0.3746–0.5880
Cd5	14.861 13.726–17.315	1.3960 1.1715–1.8040	0.1336 * 0.1010–0.1640 ↑ 21%	0.8530 *** 0.7591–0.9941 ↓ 21%	0.4650 0.3731–0.5843
Cd5 + AE	15.757 13.524–18.984	1.3219 1.2104–1.6073	0.1355 * 0.1213–0.1560 ↑ 22%	1.3779 *** ^††† ‡‡‡^ 1.2279–1.5017 ↑ 28% 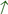 61%	0.4770 0.3574–0.6427
**10 Months**					
Control	15.968 12.280–19.566	0.9707 0.8403–1.2758	0.0885 0.0777–0.1260	1.0730 0.9609–1.2735	0.4818 0.4029–0.5079
AE	16.516 13.132–21.623	0.9972 0.8799–1.1366	0.0906 0.0728–0.1045	0.9985 0.6901–1.4094	0.4307 0.3573–0.6381
Cd1	17.523 15.420–18.591	1.0433 0.8891–1.2831	0.0960 0.0656–0.1057	0.9387 * 0.6052–1.0903 ↓ 12%	0.5099 0.3833–0.5852
Cd1 + AE	16.465 13.524–18.433	0.9824 0.9333–1.0921	0.0901 0.07373–0.1465	1.2195 ^††^ 0.9981–1.4677 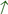 30%	0.4877 0.3532–0.6908
Cd5	21.047 *** ^‡‡‡^ 16.154–23.227 ↑ 32%	1.1004 1.0737–1.1784	0.1007 0.0779–0.1376	1.2202 ^‡^ 0.8001–1.4410	0.5897 * 0.4779–0.6411 ↑ 22%
Cd5 + AE	16.355 ^†††^ 13.607–17.993 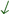 22%	1.0725 0.9453–1.1966	0.0910 0.0725–0.1204	0.9244 ^† ‡‡^ 0.7660–1.1211 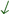 24%	0.4705 0.4010–0.6262

^3^ Data represent the median and minimum and maximum values for eight rats (except for seven animals in the AE, Cd1, and Cd5 groups after 24 months). Statistically significant differences: * *p* < 0.05, ** *p* < 0.01, *** *p* < 0.001 vs. control group; ^†^ *p* < 0.05, ^††^ *p* < 0.01, ^†††^ *p* < 0.001, vs. respective group receiving Cd alone (Cd1 or Cd5 group); ^‡^ *p* < 0.05, ^‡‡^ *p* < 0.01, ^‡‡‡^ *p* < 0.001 vs. respective group receiving the 1 mg Cd/kg diet alone (Cd1 group) or with the AE (Cd1 + AE group) are marked. ^4^ Percentage change compared to the control group (↓, decrease; ↑, increase) or the respective group receiving Cd alone (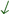, decrease; 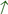, increase).

**Table 4:** The total content of Mn in the internal organs in particular experimental groups after 17 and 24 months of the study ^3^.

Group	Mn Content (μg)				
	Liver	Kidneys	Spleen	Brain	Heart
17 Months					
Control	19.986 17.953–24.879	1.1942 1.1215–1.4911	0.1492 0.1239–0.1849	1.1898 0.9564–1.6245	0.5696 0.4657–0.7360
AE	19.854 18.933–24.663	1.3243 1.1184–1.5192	0.1919 * 0.1411–0.2750 ↑ 29% ^4^	1.1590 1.0233–1.4371	0.6174 0.5222–0.7383
Cd1	19.610 16.339–27.141	1.2306 0.6885–1.4514	0.1390 0.1094–0.1982	1.1355 0.8848–1.5478	0.4915 0.4441–0.5860
Cd1 + AE	21.565 16.131–23.630	1.3745 0.8566–1.5348	0.1780 0.1411–0.2330	1.1292 0.9455–1.1983	0.5181 0.4236–0.7106
Cd5	19.194 15.017–24.167	1.3954 ^‡^ 1.2463–1.5796	0.1597 0.1413–0.1960	0.9732 ** ^‡^ 0.7127–1.1645 ↓ 18%	0.5680 0.3973–0.7040
Cd5 + AE	18.230 15.648–29.830	1.4082 1.2262–1.5270	0.1473 ^‡^ 0.1160–0.1893	1.1166 0.9020–1.3074	0.5094 0.4084–0.6568
24 Months					
Control	20.502 18.387–26.985	1.7338 1.1643–1.9272	0.1882 0.1527–0.2158	1.2136 1.1322–1.3682	0.8048 0.5802–0.8828
AE	21.714 18.427–26.752	1.5159 1.1668–2.2083	0.1823 0.1333–0.3042	1.4236 0.8137–1.4560	0.6920 0.5522–0.9004
Cd1	21.173 16.252–25.473	1.6397 1.3793–2.2194	0.1771 0.1200–0.2821	1.4519 ** 1.2269–1.8314 ↑ 20%	0.6980 0.6137–0.7732
Cd1 + AE	24.604 19.232–29.978	1.8263 1.3935–2.0252	0.2148 0.1565–0.2889	1.1785 ^††^ 0.8500–1.6496 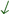 19%	0.6550 0.5887–0.7678
Cd5	29.789 *** ^‡‡‡^ 24.711–34.724 ↑ 45%	2.1949 ** ^‡^ 1.6741–2.3331 ↑ 27%	0.2125 0.1854–0.2678	1.3887 1.1759–1.6099	0.6304 * 0.5377–0.6879 ↓ 22%
Cd5 + AE	20.619 ^†††^ 13.679–28.991 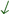 31%	1.7185 ^†^ 1.2205–2.0305 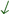 22%	0.1852 0.1513–2800	1.2137 1.0994–1.3173	0.5343 *** ^† ‡^ 0.4607–0.6713 ↓ 34% 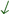 15%

^3^ Data represent the median and minimum and maximum values for eight rats (except for seven animals in the AE, Cd1, and Cd5 groups after 24 months). Statistically significant differences: * *p* < 0.05, ** *p* < 0.01, *** *p* < 0.001 vs. control group; ^†^ *p* < 0.05, ^††^ *p* < 0.01, ^†††^ *p* < 0.001, vs. respective group receiving Cd alone (Cd1 or Cd5 group); ^‡^ *p* < 0.05, ^‡‡‡^ *p* < 0.001 vs. respective group receiving the 1 mg Cd/kg diet alone (Cd1 group) or with the AE (Cd1 + AE group) are marked. ^4^ Percentage change compared to the control group (↓, decrease; ↑, increase) or the respective group receiving Cd alone (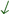, decrease).

## Data Availability

The data presented in this study are available on request from the corresponding authors. The data are not publicly available.

## Supplementary Materials

The following are available online at https://www.mdpi.com/article/10.3390/nu14224773/s1, Table S1: Analytical quality of the measurements of Mn and Cd in certified reference materials; Table S2: The daily intake of Mn with diet in particular experimental groups during the 5-day balance study; Table S3: The daily intake of Mn with 0.1% AE; Table S4: The impact of AE on the body retention of Mn in the rats exposed to Cd; Table S5: The impact of AE on the total body burden of Mn in rats exposed to Cd.

## Abbreviations

AAS method, atomic absorption spectrometry; Abs_Mn_, apparent absorption of manganese during the 5-day balance study; AE, extract from the berries of *Aronia melanocarpa* L.; *A. melanocarpa*, *Aronia melanocarpa* L.; b.w., body weight; Cd, cadmium; CdCl_2_, cadmium chloride; Cd^2+^, cadmium ion; Cu, copper; CV, coefficient of variation; FE_Mn_, mean amount of manganese daily excreted with faeces during the 5-day balance study; GF AAS, flameless atomic absorption spectrometry with electrothermal atomization in a graphite furnace; HCl, hydrochloric acid; HNO_3_, nitric acid; H_2_O, water; H_2_O_2_, hydrogen peroxide; I_Mn_, mean daily intake of manganese via food during the 5-day balance study; *i.p.*, intraperitoneally; Mn, manganese; MnCl_2_, manganese chloride; MnSOD, manganese-dependent superoxide dismutase; Mn(II), divalent manganese; Mn^2+^, ion of Mn(II); MT, metallothionein; NaCl, sodium chloride; O_2_^·−^, superoxide radical; ^·^OH, hydroxyl radical; R_Mn_, mean daily body retention of manganese evaluated during the 5-day balance study; ROS, reactive oxygen species; SD, standard deviation; SE, standard error; SOD, superoxide dismutase; *s.c.*, subcutaneously; UE_Mn_, mean amount of manganese daily excreted with urine during the 5-day balance study; *v/v*, volume/volume; Zn, zinc.
