# Supplementary material for: The Body Status of Manganese and Activity of This Element-Dependent Mitochondrial Superoxide Dismutase in a Rat Model of Human Exposure to Cadmium and Co-Administration of Aronia melanocarpa L. Extract
Source: Nutrients. 2022 Nov 11;14(22):4773. doi: 10.3390/nu14224773 (PMC9699381; doi:10.3390/nu14224773)
Supplement: Supplementary file 1 [file nutrients-14-04773-s001.zip › nutrients-2002577-supplementary.pdf]

nutrients

MDPI

Supplementary Material

Supplementary Table S1. Analytical quality of the measurements of Mn and Cd in certified reference materials.

Kind of Certified Reference Material	Reference Values [1]	Noticed Values [1]	Recovery	Precision (CV) [2]
Analytical Quality of Mn Measurements				
Trace Elements Serum L-1 LOT 0903106 (SERO AS, Billingstad, Norway)	15.0 ± 0.9 µg/L	14.6 ± 0.6 µg/L	97%	4.1%
Trace Elements Urine L-2 (LOT 1011645; SERO AS, Billingstad, Norway)	10.9 ± 2.2 µg/L	10.4 ± 0.4 µg/L	95%	3.8%
Standard Reference Material Bovine Liver (no. 1577b; National Institute of Standards and Technology, Gaithersburg, MD, USA)	10.5 ± 1.7 µg/g	11.5 ± 0.4 µg/g	109%	3.5%
Certified Reference Material BCR Pig Kidney (BCR-186; Institute for Reference Materials and Measurements, Geel, Belgium)	8.5 ± 0.3 µg/g	8.3 ± 0.3 µg/g	98%	3.6%
Standard Reference Bone Ash (no. 1400; National Institute of Standards and Technology, Gaithersburg, MD, USA)	17 µg/g	16.5 ± 0.3 µg/g	97%	1.8%
Analytical Quality of Cd Measurements				
Standard Reference Material Bovine Liver (no. 1577b; National Institute of Standards and Technology, Gaithersburg, MD, USA)	0.50 ± 0.03 µg/g	0.517 ± 0.025 µg/g	103%	4.8%
Certified Reference Material BCR Pig Kidney (BCR-186; Institute for Reference Materials and Measurements, Geel, Belgium)	2.71 ± 0.15 µg/g	2.63 ± 0.17 µg/g	97%	6.5%

[1] Data are represented as mean ± SD for three measurements. [2] Precision of measurements is expressed as a CV.

Supplementary Table S2. The daily intake of Mn with diet in particular experimental groups during the 5-day balance study [3,4,5,6].

Group	Mn Intake (mg/24 h)			
	3 Months	10 Months	17 Months	24 Months
Control	3.581<br>3.379–3.756	2.310***<br>2.208–2.712	2.736***<br>2.388–2.856	3.318*<br>3.168–3.684
AE	3.567<br>3.321–3.799	2.508***<br>2.280–2.556	2.586***<br>2.388–2.796	3.204**<br>3.048–3.468
Cd1	3.539<br>3.306–3.770	2.460***<br>2.328–2.652	2.568***<br>2.400–2.736	3.372<br>3.228–3.696
Cd1 + AE	3.473<br>3.306–3.582	2.478***<br>2.268–2.736	2.514***<br>2.400–2.850	3.384<br>3.024–3.948
Cd5	3.509<br>3.364–3.698	2.490***<br>2.364–2.640	2.520***<br>2.280–2.616	3.348<br>3.180–3.972
Cd5 + AE	3.582<br>3.408–3.683	2.394***<br>2.352–2.496	2.508***<br>2.400–2.676	3.252*<br>2.952–3.840

[3] The rats received 0.1% aqueous extract from the berries of *Aronia melanocarpa* L. (AE) or not and Cd in the diet at the concentration of 0, 1, and 5 mg/kg. [4] The study was performed in the last week of the 3rd, 10th, 17th, and 24th months of the experiment. [5] The intake of Mn was calculated based on this element concentration in the Labofeed diets declared by the manufacturer. The Labofeed H diet (administered throughout the first 3 months of the study) contained 145 mg Mn/kg, whereas the Labofeed B diet (used thereafter) contained 120 mg Mn/kg. [6] Data represent a median and minimum and maximum values for 8 rats (except for 7 animals in the AE, Cd1, and Cd5 groups after 24 months). * $p < 0.05$, ** $p < 0.01$, *** $p < 0.001$ compared to the intake in the last week of the 3rd month.

Supplementary Table S3. The daily intake of Mn with 0.1% AE [7,8,9].

Group	Experiment Duration			
	**3 Months**	**10 Months**	**17 Months**	**24 Months**
	Mn Intake (μg/24 h)			
Control	-	-	-	-
AE	0.0173<br>0.0161–0.0179	0.0163<br>0.0135–0.0184	0.0164<br>0.0148–0.0174	0.0158<br>0.0150–0.0190
Cd1	-	-	-	-
Cd1 + AE	0.0169<br>0.0155–0.0183	0.0160<br>0.0149–0.0173	0.0167<br>0.0154–0.0174	0.0169<br>0.0153–0.0186
Cd5	-	-	-	-
Cd5 + AE	0.0170<br>0.0157–0.0182	0.0169<br>0.0142–0.0183	0.0169<br>0.0148–0.0181	0.0163<br>0.0139–0.0190
	Mn Intake (μg/kg b.w./24 h)			
Control	-	-	-	-
AE	0.0560<br>0.0521–0.0579	0.0376***<br>0.0313–0.0426	0.0317***<br>0.0286–0.0337	0.0278***<br>0.0264–0.0335
Cd1	-	-	-	-
Cd1 + AE	0.0563<br>0.0515–0.0608	0.0361***<br>0.0336–0.0390	0.0332***<br>0.0306–0.0354	0.0294***<br>0.0267–0.0324
Cd5	-	-	-	-
Cd5 + AE	0.0538<br>0.0497–0.0576	0.0403***<br>0.0339–0.0437	0.0335***<br>0.0292–0.0358	0.0294***<br>0.0251–0.0343

[7] The intake of Mn was calculated based on this element concentration determined in the 0.1% AE (0.396 ± 0.039 μg/L). [8] Data represent a median value and minimum and maximum intake of Mn for 32, 24, 16, and 8 rats during 3, 10, 17, and 24 months, respectively, except for 7 animals in the AE group in the last time-point of the experiment. *** $p < 0.001$ compared to the intake during 3 months. [9] The intake of Mn in the control, Cd1, and Cd5 groups was recognized to be 0.

Supplementary Table S4. The impact of AE on the body retention of Mn in the rats exposed to Cd [6].

	Mn Body Retention (%)			
**Group**	**3 Months**	**10 Months**	**17 Months**	**24 Months**
Control	57.590	55.923	54.248	56.263
	55.104–61.262	47.055–58.113	48.646–63.559	52.241–59.065
AE	58.088	49.423	53.592	55.714
	53.098–61.114	44.458–59.769	47.905–61.362	50.317–61.110
Cd1	49.974***	47.263*	56.841	57.981
	44.834–52.735	40.075–57.098	46.983–64.791	56.642–58.666
Cd1 + AE	51.466***	47.766	56.119	57.361
	46.298–55.527	44.879–59.794	46.444–61.190	52.809–64.792
Cd5	49.339***	61.811** ‡‡‡	50.007 ‡	65.156*** ‡‡
	41.513–52.998	55.796–69.270	47.084–57.491	57.870–67.279
Cd5 + AE	54.097** †	56.887† ‡‡	52.266	58.268†
	48.210–55.610	51.496–65.481	45.809–58.865	50.846–66.432

[6] Data represent a median and minimum and maximum values for 8 rats (except for 7 animals in the AE, Cd1, and Cd5 groups after 24 months). Statistically significant differences: * $p < 0.05$, ** $p < 0.01$, *** $p < 0.001$ vs. control group; † $p < 0.05$ vs. Cd5 group; ‡ $p < 0.05$, ‡‡ $p < 0.01$, ‡‡‡ $p < 0.001$ vs. respective group receiving the 1 mg Cd/kg diet alone (Cd1 group) or with the AE (Cd1 + AE group) are marked.

Supplementary Table S5. The impact of AE on the total body burden of Mn in rats exposed to Cd [6].

Group	Total Body Burden of Mn (µg)			
	**3 Months**	**10 Months**	**17 Months**	**24 Months**
Control	20.440<br>16.795–22.838	18.680<br>14.769–22.563	23.040<br>21.237–28.332	24.264<br>21.475–32.024
AE	19.034<br>17.579–20.051	19.056<br>15.448–24.511	23.380<br>22.067–27.885	25.850<br>21.954–30.949
Cd1	17.982<br>16.193–29.498	19.997<br>18.041–21.114	22.897<br>18.864–30.315	25.280<br>20.304–29.516
Cd1 + AE	18.647<br>15.353–21.400	19.206<br>16.060–21.558	24.886<br>18.984–26.749	28.400<br>23.198–33.592
Cd5	17.630<br>16.705–20.074	23.873$^{***\ ‡‡‡}$<br>18.831–26.297<br>↑ 28% [10]	22.620<br>17.630–26.491	36.020$^{***\ ‡‡‡}$<br>29.023–39.095<br>↑ 48%
Cd5 + AE	19.078<br>16.945–22.298	19.240†††<br>17.007–20.611<br>↓ *19%*	21.351<br>18.399–32.971	22.432$^{†††\ ‡}$<br>17.731–32.619<br>↓ *38%*

[6] Data represent a median and minimum and maximum values for 8 rats (except for 7 animals in the AE, Cd1, and Cd5 groups after 24 months). Statistically significant differences: *** $p < 0.001$ vs. control group; ††† $p < 0.001$ vs. Cd5 group; ‡ $p < 0.05$, ‡‡‡ $p < 0.001$ vs. respective group receiving the 1 mg Cd/kg diet alone (Cd1 group) or with the AE (Cd1 + AE group) are marked. [10] Percentage change compared to the control group (↑, increase) or the respective group receiving Cd alone (↓, decrease).
